# Cross-reactive tissue-resident memory T lymphocytes—concepts, evidence, and open questions

**DOI:** 10.1186/s12967-026-08137-7

**Published:** 2026-05-07

**Authors:** Hong Lei, Ying Sun, Weihua Gong, Jun Dong

**Affiliations:** 1 Key Laboratory of Precision Medicine to Pediatric Diseases of Shaanxi Province, Shaanxi Institute for Pediatric Diseases, Xi’an Children’s Hospital, Affiliated Children’s Hospital of Xi’an Jiaotong University, Xi’an, 710003 China; 2 Department of Surgery, Second Affiliated Hospital of School of Medicine, Zhejiang University, Hangzhou, 310058 China; 3 Cell Biology, Deutsches Rheuma-Forschungszentrum Berlin (DRFZ), An Institute of the Leibniz Association, Berlin, Germany

**Keywords:** Tissue-resident memory T cells, Cross-reactivity, Cancer, Autoimmunity, Demethylation

## Abstract

**Background:**

Tissue-resident memory T (Trm) cells have emerged as a distinct lymphocyte lineage that provides rapid, frontline immune surveillance in peripheral tissues while also contributing to durable systemic immunity. A critical and evolving aspect of Trm cell biology is their inherent cross-reactivity—the ability of a single T cell receptor (TCR) to recognize and respond to multiple related or unrelated antigens beyond its primary target.

**Main body:**

This review synthesizes current knowledge on the dual roles of cross-reactive Trm cells, which can mediate broad protection against heterologous infections and cancers but may also precipitate or exacerbate autoimmune pathology. We first address the developmental origins, tissue-specific distribution (from barrier surfaces to internal organs, including the central nervous system and bone marrow niches), and key functions that define the Trm population. A central focus is placed on the mechanistic basis of TCR cross-reactivity, comparing and contrasting its regulation in circulating memory T cells versus Trm cells. We delve into how tissue-specific signals, particularly local cytokine milieus and epigenetic reprogramming such as demethylation imprints, shape the functional avidity and antigenic breadth of Trm cell responses. Finally, we discuss emerging therapeutic strategies designed to either harness the protective cross-reactivity of Trm cells for next-generation vaccines and immunotherapies or to restrain its detrimental potential in autoimmune and inflammatory diseases.

**Conclusions:**

Trm cells are powerful immune sentinels whose TCR cross-reactivity enables broad protection but also risks autoimmunity. Understanding these precise rules is key to developing Trm-based immunotherapies against cancer and infections while avoiding autoimmunity.

**Supplementary Information:**

The online version contains supplementary material available at 10.1186/s12967-026-08137-7.

## Introduction

T cells are central to adaptive immunity, capable of mounting powerful antigen-specific responses. The establishment of immunological memory ensures that these responses are faster and more robust upon re-exposure to pathogens. This memory is embodied by specialized, long-lived memory T cells, which can be broadly categorized into circulating and tissue-resident populations. Circulating memory T cells include central memory (Tcm) and effector memory (Tem) T cells [1–3]. Tcm primarily reside in and recirculate through secondary lymphoid organs, where they provide durable protection, whereas Tem patrol peripheral tissues to exert immediate effector functions. However, the protective capacity of these circulating subsets in peripheral tissues can be transient (weeks to months). Consequently, long-term immunity is now recognized to rely heavily on tissue-resident memory T (Trm) cells, which are permanently lodged within tissues to act as local sentinels and/or systemic reservoirs [4, 5].

The classical paradigm of Trm cells casts them as frontline defenders restricted to barrier sites like the skin and mucosal surfaces. However, it is now evident that Trm cells also reside in non-barrier tissues, including the bone marrow, liver, salivary glands, and brain, these Trm populations appear to function as long-lived reservoirs that contribute to durable, body-wide protection. The bone marrow, in particular, provides a unique niche that maintains Trm cells with enhanced stem-like potential and longevity. Although non-circulating under steady-state conditions, bone marrow Trm cells can also be mobilized upon reactivation and contribute to secondary systemic immune responses [6–8]. The major shared surface marker for Trm cells across different organs is CD69, while CD103 is expressed more variably and mainly in CD8^+^ Trm cells in epithelial sites and liver [9, 10]. Additional surface markers like CXCR6, CD49a and PD-1 are also reported to be expressed by Trm cells in certain organs, such as bone marrow, lung, liver, and the central nervous system (CNS) [11–14]. Recent studies suggest tissue CD69- memory T cells could be also tissue resident [15, 16]. To date, Trm populations have been identified across various barrier tissues as well as primary (bone marrow) [17–21] and secondary (spleen, lymph nodes) [22, 23] lymphoid organs.

A critical functional property that significantly influences the efficacy of Trm cells is the cross-reactivity of their T cell receptors (TCRs). Cross-reactivity is the ability of a TCR to recognize and respond to multiple different peptide-MHC (Major Histocompatibility Complex) complexes, not just the one it was originally primed against. This intrinsic feature of TCR recognition allows the immune system to cover vast antigenic diversity with a limited TCR repertoire, thereby providing broad-spectrum and heterologous immunity against evolving pathogens [24]. However, this same capacity can be detrimental when T cells cross-react with self-antigens or environmental allergens, potentially driving autoimmune diseases or allergies. Similarly, alloreactive T cells that cross-react with donor antigens are a major driver of transplant rejections. Therapeutically, this double-edged sword can be harnessed; vaccination strategies aimed at generating cross-reactive T cells against conserved epitopes, such as those in flu, can induce broad protection across viral strains [25, 26]. In this review, we’ll focus on the role of cross-reactive Trm populations across different anatomical sites in health and diseases. Due to space constrains, this review is not exhaustive, and we refer readers to the broader literature for addtional studies.

## Origins, tissue distribution, and functional niches of Trm cells

**Fig. 1:**
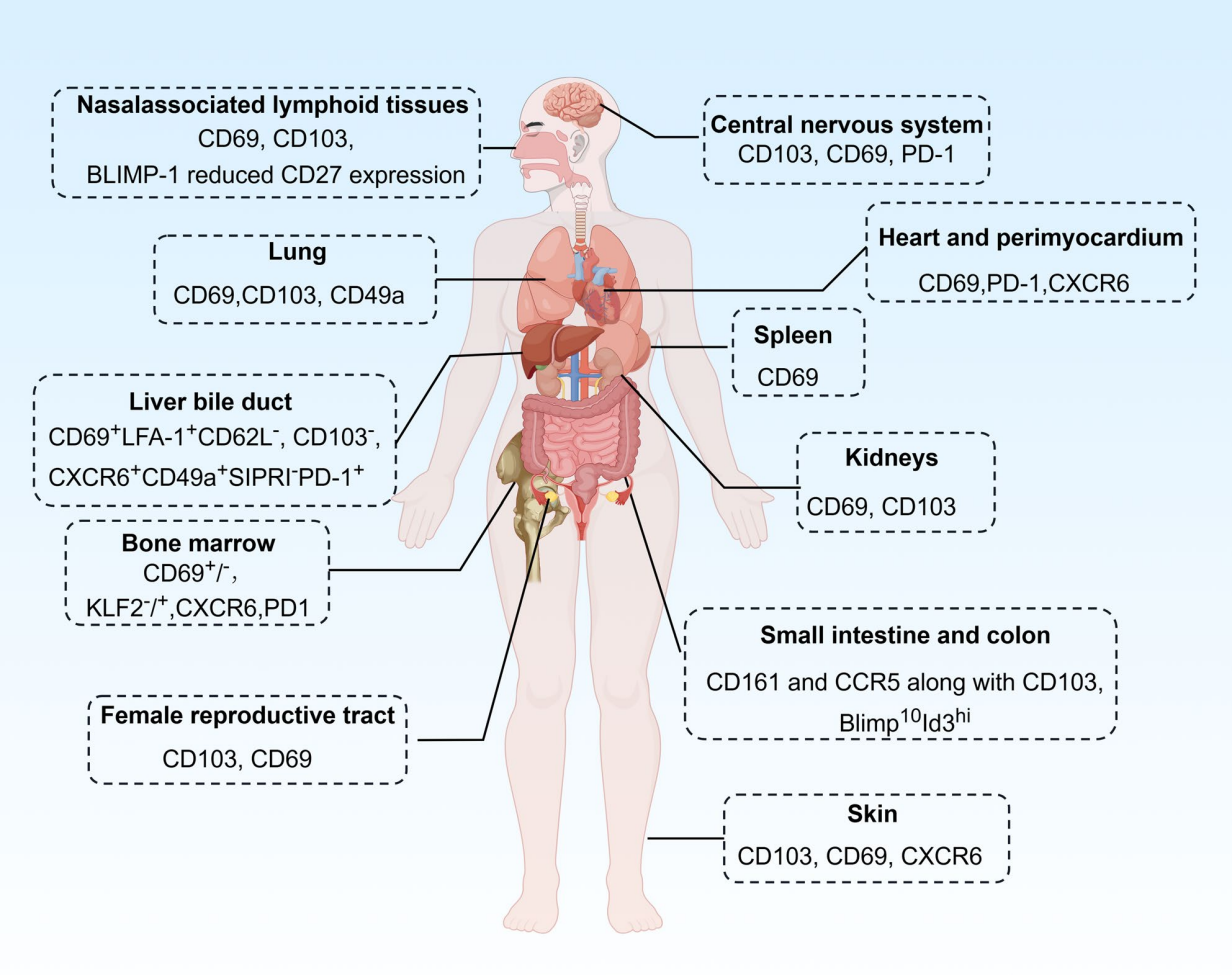
Distribution and phenotype of Trm cells at different locations. Schematic diagram was created using Figdraw2.0

Naive T cells get activated when they encounter their specific antigen presented by MHC molecules on antigen-presenting cells. Once activated, they proliferate and differentiate into effector T cells and migrate to the site of infection to combat the pathogen. After the infection is cleared, a small subset of T cells survives and differentiates into memory T cells [27]. When effector T cells are activated during infection or inflammation in peripheral tissues, they gradually develop into Trm cells. In the early 1960s, Gowans and McGregor conducted groundbreaking experiments using thoracic duct drainage to study lymphocyte biology and immune memory, and demonstrated that secondary (memory) immune responses are not affected by depletion of all circulating lymphocytes [28]. Since then, Trm cell compartments have further been delineated through multiple approaches, including confinement in parabiosis experiments [15, 29], distinct tissue-specific gene marker expression, such as CD69 [17, 21, 23, 30], and tissue-tailored antigen receptor repertoires and specificities [17, 21, 31]. To date, Trm populations have been found across many different sites such as the skin [32], bone marrow [17, 18, 21], spleen [33], heart [34, 35], liver [9, 36], kidney [33], nasal mucosal tissue [37], gut [38], lungs [38, 41–43], female reproductive tract [40] and brain [14, 41] (see Fig. 1). Together, these studies are complemented by an overview of phenotypic markers, functional characteristics, and methods used to identify Trm populations across different tissues (Table 1).

**Table 1:** Characteristics and cross-reactivities of Trm cells across anatomical sites

Distribution/ Locations	Marker epitopes	Functions	Methods of discovery	Cross reactivities
Skin [42]	CD103, CD69, CXCR6	ϑ Rapid pathogen response and monitoring melanoma cells; Λ drive chronic inflammation in conditions like psoriasis	Tissue grafting models and phenotypic and quantatitive analyses [43, 44]	drug antigen reactive skin Trm cells killed surrounding epidermal cells, resulting in drug-induced skin lesions [45, 46]
Bone marrow [16, 17, 21]	CD69, CXCR6, KLF2	Confer not only bone marrow, but also systemic immune memory [8, 47]	Compare human memory T cells in blood and bone marrow on proliferation, transcription, mobility and repertoire.	Tetanus toxoid-reactive Trm cellscross-react with the measles-mumps-rubella (MMR) vaccine [8]; Pre-existing SARS-CoV-2-reactive memory T cells in unexposed individuals [48]; Cross-reactive to virus like SARS-CoV-2 [49]
(Mucosa) Nasal-associated lymphoid tissues (NALT) [37]	CD69, CD103, and BLIMP-1 reduced CD27 expression	Protect against SARS-CoV-2 infection [49]; cytotoxic activity and cytokine production ex vivo, but failed to control EBV viral loads in the NALT during infection	Using a symptomatic primary EBV infection model in mice with a humanized immune system	
Spleen [22, 23, 50]	CD69	Protect against viral infections	Human tissue by core transcriptional and functional signatures [22, 23]	iIntrasplenic CD8 Trm cells cross-reactive to NS31629-1637 protein of HCV stains [51]
Lung [39, 52, 53]	CD69, CD103, CD49a	ϑ provide immunity against Klebsiella and influenza; prevent tumor lung metastasis Λ alloreactive TCRs in both graft-versus-host and host-versus-graft after lung transplantation	intravital imaging in mice and TCRβ-sequencing of human lung tissue	Lung Trm cells contained recipient-derived repertoire and multi-tissue-shared TCRs after lung Tx
Heart and perimyocardium [34, 35]	CD69, PD-1, CXCR6	Λ increase susceptibility to immune checkpoint inhibitor-associated-myocarditis	Using a modified experimental autoimmune myocarditis mouse model and an ischemia-reperfusion injury mouse model	Cardiac myosin-specific (MyHC) reactive Trm cells
Small intestine and colon [38]	CD161 and CCR5 along with CD103 Blimp1^lo^Id3^hi^	Λ Contribute to Crohn’s disease [54]	next generation sequencing to serial blood, lymphoid tissue, and gut samples of human	alloreactive and microbe-reactive repertoire in intestine transplant setting [55]
Liver [9, 56] bile duct [57]	CD69^+^LFA-1^+^CD62L^−^ CD103^−^ CXCR6^+^CD49a^+^S1PR1^−^PD-1^+^	Rapid clearance of RHV reinfection; Protect against HCV infection in liver Autoimmune liver disease [57]	rodent HCV-like virus (RHV) infections in mice; RNA-seq on bile of patients with PSC and non-PSC controls	intrahepatic CD8 Trm cells cross-reactive to NS31629-1637 protein of HCV stains [51]
Kidneys [33]	CD69 and CD103	Kidney Trm cells drive rejection in a kidney transplantation model [58]	Using a kidney transplantation model	Cross reactivity to recipient antigens
Central nervous system (CNS) [14, 59, 60]	CD69 and CD103 PD-1	Against local infections and local autoantigens in driving neuroinflammation	Using mice with LCMV infection; mice model of Alzheimer’s disease	
Female reproductive tract [40]	CD69 and CD103	antiviral defense to sexually transmitted infections	single-cell RNA-Seq approaches to define gene expression and TCR clonotypes of the human ectocervix	TCR repertoires target anatomically relevant viral antigens

The table summarizes the defining features of Trm cells inhabiting distinct human tissues. Rows are organized by anatomical distribution. For each site, the corresponding Trm population is detailed through five key attributes: their distribution/locations within the tissue; canonical surface and functional Marker profiles; primary immunological functions in local surveillance and response; experimental methods of discovery used for their identification and study; and documented cross-reactivities, indicating their recognized target antigens or potential for heterologous immunity

Trm cells development in certain tissues is regulated by key cytokines such as TGF-β, IL15 and type I Interferons (IFN-α/β). TGF-β promoted Trm differentiation by upregulating adhesion molecules (e.g., CD103), and IL-15 supported Trm survival and maintenance in tissues while type I Interferons (IFN-α/β) enhanced Trm formation in mucosal sites [5, 61, 62]. Trm cells adapt in an organ-specific manner and show diverse phenotypes depending on the local tissue microenvironments (Table 1). For instance, skin Trm cells localize to the epidermis and dermis, adhere to epithelial cells via E-cadherin with expression of CD69 and CD103 [32]; gut CD4^+^ and CD8^+^ Trm cells also express CD103 and CD18 to retain in the intestinal epithelium and lamina propria. Trm cells residing in lung often express CXCR6 to respond to chemokine receptors like CXCL16, which depend on TGF-β and IL-15 for survival in the oxygen-rich lung niche [63]. On the other hand, both CD69^−^ and CD69^+^ Trm subsets have been identified in the bone marrow [16, 17, 50], with the latter associating with IL-7 expressing stromal cells, presumably providing maintenance signals [18, 21].

However, animal studies showed that former Trm cells can also rejoin the circulation, although the exact tissue origin was not clear [6, 7] On the other hand, Trm cells in human bone marrow can be mobilized and contribute to systemic secondary immune reactions [8]. Interestingly, recent findings showed that the chemokines like CCL3, CCL4 and CXCL10 on Trm cells also attract neutrophils, monocytes, NK cells, and effector T cells by binding receptors to amply the inflammation at local sites [64, 65]. Trm cells also exhibit substantial functional heterogeneity. CD8⁺ Trm cells target enteric viruses such as rotavirus and bacteria pathogens including salmonella, whereas CD4⁺ Trm cells regulate antifungal immunity, for example in response to *Candida* infections [66]. CD8⁺ Trm cells can rapidly secrete IFN-γ and granzyme B against viruses like influenza and other infections, while CD4⁺ Trm cells in asthma contribute to eosinophil recruitment and airway remodeling by driving IL-5/IL-13 production [67, 68, 125].

While the developmental cues, maintenance requirements, and functional heterogeneity of Trm cells are increasingly recognized, these features are also epigenetically imprinted. DNA methylation provides a durable framework that both diversifies functional potential [69] and secures long-term persistence. For example, we recently found that tissue-specific demethylation at *CCL5* (RANTES), *IL13*, *CSF2* (encoding GM-CSF), and *PFR1* (encoding perforin) supports rapid recall responses, whereas epithelial tissue-specific demethylation at *ITGAE* (encodes CD103) and *ITGB2* (encodes CD18) reinforces survival and niche interactions [70]. Together, these imprints ensure the durable identity and function of Trm cells.

## Cross-reactivity in circulating T cells

**Fig. 2:**
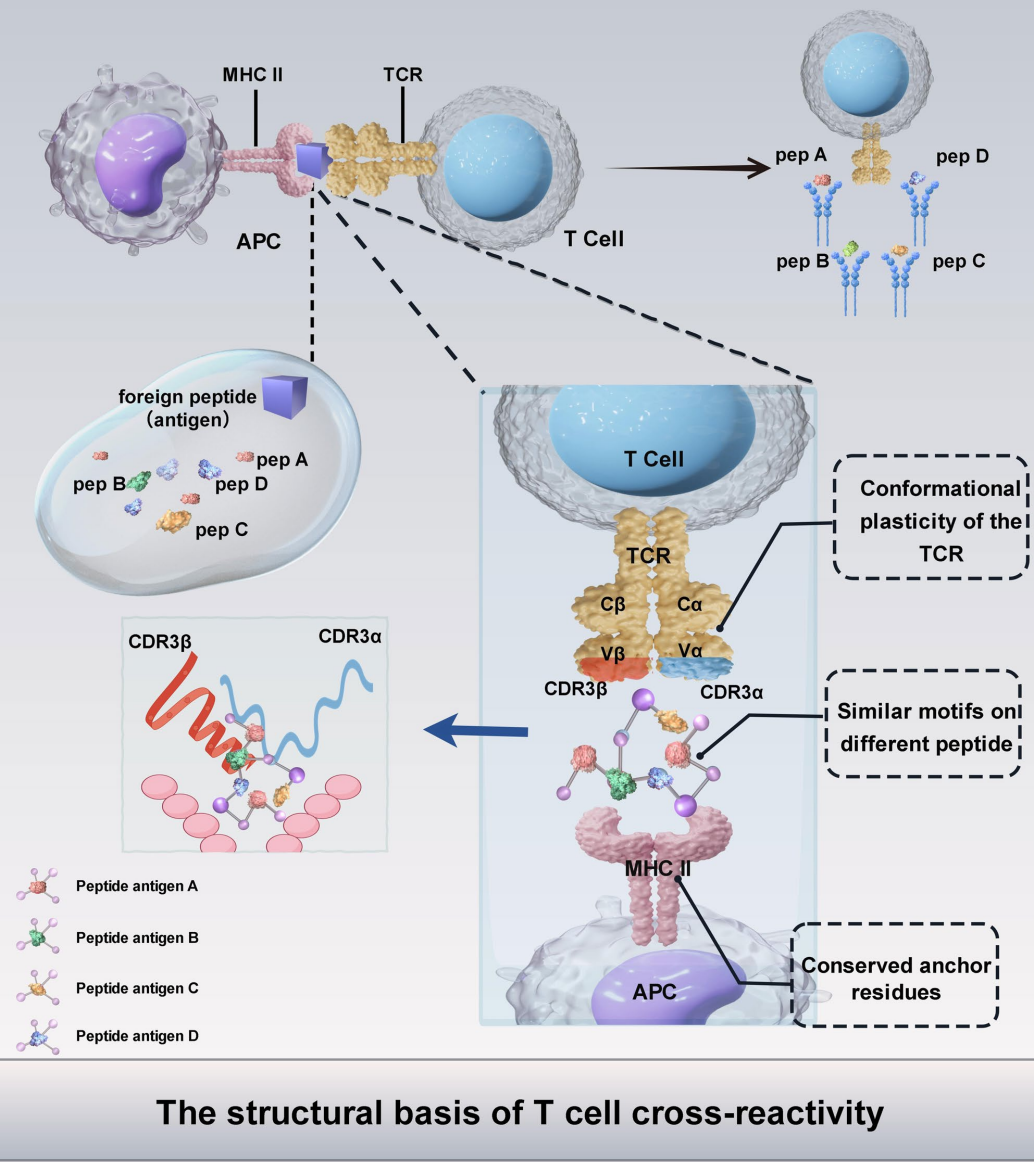
Mechanistic model of TCR cross-reactivity driven by CDR3 loop flexibility. The diagram illustrates how a single T cell receptor (TCR), specific for a foreign peptide (purple), can cross-react with a set of distinct self- or pathogen-derived peptides (A-D; orange, green, yellow, blue). This promiscuity arises from the recognition of shared or similar molecular motifs between antigens, enabled by the intrinsic flexibility and adaptability of the highly variable CDR3 loops. The CDR3α is highlighted with light blue and CDR3β is highlighted with orange in the TCR-Ag-MHC binding model, with additional enlarged schematic diagram of the binding complex on the left. Schematic diagram was created using Figdraw2.0

Cross-reactivity allows a single T cell receptor (TCR) to recognize multiple different antigens or peptides presented by MHC molecules, therefore provides broad-spectrum and heterologous immunity against pathogens. The structural basis of T cell cross-reactivity arises from the interplay between the flexible binding regions of TCR and shared molecular motifs among antigens as showed in Fig. 2. The TCR structure basis for this mainly contains the following aspects: (i) the complementarity determining regions (CDRs) of the TCR, especially CDR3 loops, are highly variable and flexible. These loops allow conformational adjustments to contact with the antigen-MHC complex [71]; (ii) the peptides presented by MHC have similar motifs, like certain amino acids in key positions that the TCR can recognize even if the rest of the peptide varies [72]; (iii) the MHC molecule itself has conserved anchor residues, which enables different peptides to bind similarly [73, 74]; (iv) conformational plasticity of the TCR upon binding allows TCR to accommodate different peptides [75]. Thus, TCR cross-reactivity is rooted in the flexibility of CDR3 loops, the presence of shared molecular motifs in antigens and conformational plasticity with adaptability.

T cell cross-reactivity is essential for broad immune coverage, which enables limited TCR repertoires to mount responses against variant or even unrelated pathogens [76]. Su et al. found an abundance of memory-phenotype cells (20%-90%, averaging over 50%) of CD4^+^ T cells specific to viral antigens (such as HIV) in adults who had never been infected, which was explained as TCR cross-reactivity to environmental antigens[77]. T cell cross-reactivity plays a critical role in combating rapidly mutating viruses such as influenza and SARS-CoV-2, where conserved epitopes are targeted [25, 78]. SARS-CoV-2-reactive CD4^+^ T cells have been reported in unexposed individuals, which suggest a preexisting cross-reactive T cell memory in 20 to 50% of people [79, 80]. For instance, SARS-CoV-2-specific CD4^+^ and CD8^+^ T cell responses can originate from cross-reactive cytomegalovirus (CMV)-specific T cells [81]. T cell responses to SARS-CoV-2 spike also cross-recognize Omicron variant (B.1.1.529), which has multiple spike protein mutations and contribute to viral escape from antibody neutralization to reduce the vaccine protection from infection [82]. Therefore, vaccines can be designed to include cross-reactive epitopes to provide broad protection, such as universal flu vaccines targeting conserved viral regions. Nanoparticle-based vaccines and personalized immunotherapy approaches leverage T cell cross-reactivity as well [83].

Cross-reactive T cells in transplant recipients contribute to the development of transplant rejections [84, 85], while cross-reactive donor-specific CD8^+^ Tregs are also reported to efficiently prevent transplant rejection [86]. Other risk of off-target responses leading to misdirected bystander activation or excess immunity like autoimmunity. HLA-DR15 molecules are recently reported to jointly shape an autoreactive T cell repertoire in multiple sclerosis [87]. Autoantigen cross-reactive environmental antigen can also trigger multiple sclerosis-like disease [88].

## Cross-reactive Trm cells: double-edged roles in immunity

The strategic positioning and long-term persistence of Trm cells make their antigenic specificity a critical determinant of immune outcomes. When Trm recognize shared epitopes across disparate threats, this cross-reactivity can be therapeutically harnessed but can also precipitate pathological autoimmunity, rendering them a double-edged sword.

The clinical significance of Trm cell cross-reactivity is particularly evident in cancer. A compelling body of evidence now links intratumoral Trm cells, particularly the CD103⁺ subset, to improved patient survival in cancers like melanoma [89]. The mechanistic underpinning of this favorable prognostic extends beyond tumor-specific responses to include “heterologous immunity”, where pre-existing anti-viral memory is co-opted for cancer surveillance. This paradigm is supported by several key findings. Pioneering mouse models demonstrated that virus-specific T cells can infiltrate tumors and be repurposed for tumor immunotherapy [90]. In humans, in non-small cell lung cancer (NSCLC), tumor-infiltrating Trm cells were found to possess TCRs capable of recognizing both viral antigens (e.g., influenza, EBV, CMV) and tumor-associated antigens, directly linking a patient’s pre-existing antiviral immunity to anti-tumor responses [91]. This is complemented by the discovery of abundant “bystander” virus-specific CD8⁺ T cells within human tumors, many displaying Trm cell-like features, which are clonally expanded yet lack specificity for canonical tumor neoantigens [92]. The therapeutic exploitation of this cross-reactivity is also emerging. Intratumoral administration of a seasonal influenza vaccine can remodel the tumor microenvironment, converting immunologically “cold” tumors into “hot” ones in a process dependent on pre-existing virus-specific T cells [93]. Beyond whole vaccines, sophisticated antibody-based approaches have been designed to deliver viral epitopes directly to tumors, sensitizing them for destruction by pre-existing virus-specific T cells [94]. The principle has been further extended using live-attenuated vaccines like MMR (measles, mumps, rubella) to redirect established virus immunity into potent anti-tumor responses [95, 96]. The high frequency of these responses is explained by structural biology studies, which have identified viral antigens with significant homology to tumor-associated antigens, providing a physical basis for the observed TCR cross-reactivity [97]. The cross-reactive arsenal is not limited to CD8^+^ T cells. Evidence reveals that CD4⁺ Trm cells can also acquire potent cytotoxic activity and contribute to tumor control, suggesting a broader role for helper T cells in anti-tumor immunity [98, 99].

However, as the cornerstone of sustained antitumor immunity, the generation and function of memory T cells are profoundly shaped by the tumor microenvironment (TME), including interactions with tumor-associated macrophages (TAMs). Macrophage polarization states can influence T cell activation and persistence within tissues, thereby modulating the functional capacity of Trm cells. Recent studies suggest that such microenvironmental factors may impact responses to checkpoint inhibitors (ICIs) targeting PD-1/PD-L1 (e.g. pembrolizumab, atezolizumab) or CTLA-4 (e.g. ipilimumab) [100, 101]. However, the extent to which cross-reactive Trm populations contribute to these processes remains incompletely understood.

Conversely, the same mechanistic principle of cross-reactivity underpins one of the central hypotheses in autoimmunity: molecular mimicry. Foundational studies in experimental autoimmune encephalomyelitis (EAE), a model for multiple sclerosis, established that viral infections can trigger T-cell responses against self-antigens in the central nervous system [102]. For instance, infections with Theiler’s murine encephalomyelitis virus were shown to activate T cells that cross-react with myelin epitopes, thereby exacerbating disease. This demonstrates how a peripheral viral infection can, through cross-reactive Trm cells or other memory populations, ignite and sustain tissue-specific autoimmune [103, 104]. More broadly, infections such as lymphocytic choriomeningitis virus (LCMV) have been shown to profoundly alter immune responses to subsequent unrelated pathogens in both mice and human a phenomenon known as “heterologous immunity” that can sometimes have deleterious consequences [105, 106]. Together, these studies highlight the dark side of TCR degeneracy, wherein cross-reactive Trm memory T cells, including long-lived Trm cells, can become inadvertent executioners of healthy tissue.

## Tissue-specific cues and methodological advances in defining cross reactive Trm cells

The functional outcome of Trm cell cross-reactivity is profoundly shaped by the tissue microenvironment, which dictates their differentiation, metabolic state, and antigenic experience. Concurrently, cutting-edge technologies are now providing an unprecedented view into the clonal dynamics and spatial organization of these cells.

The local milieu imposes distinct functional programs on Trm cells, directly their antigenic breadth. Tissue-derived signals, including cytokines and persistent infections, are instrumental in driving Trm differentiation and shaping their TCR repertoire [107, 108]. Furthermore, metabolic cues such as nutrient availability and hypoxia within tissues fine-tune Trm cell fitness and effector capacity [109]. The environmental programming is exemplified in organs with constant antigen exposure. For instance, liver Trm cells develop broad antigen specificity, a likely adaptation to the continuous influx of gut-derived microbial products [110]. This phenomenon, however, can have deleterious consequences, as demonstrated by donor-derived CD8^+^ Trm cells that mediate gastrointestinal acute graft-versus-host disease (GvHD) following allogeneic hematopoietic stem cell transplant (allo-HCT) [64], highlighting how resident memory can drive pathology through misplaced cross-reactivity. The protective potential of environmentally imprinted Trm cells is evidenced by vaccination strategies. Immunization with a single-cycle influenza virus vaccine candidate (S-FLU) induces the deposition of nucleoprotein (NP)-specific CD8^+^ Trm cells in the respiratory tract, which are capable of recognizing diverse viral variants [26], demonstrating that localized vaccination can establish a frontline, cross-reactive defense.

Emerging evidence challenges the dogma of Trm cells as irreversibly sessile, revealing a dynamic dimension to their biology. Our recent work demonstrates that tetanus toxoid-reactive CD4⁺ Trm cells can egress from the human bone marrow into the circulation following MMR vaccination, indicating that heterologous immune challenge can mobilize pre-existing, cross-reactive Trm pools from systemic reservoirs [8]. Supporting the relevance of these pools, we have detected preexisting SARS-CoV-2–reactive CD4⁺ Trm cells in the bone marrow of unexposed individuals [48]. These findings posit the bone marrow as a central archive for, cross-reactive Trm cells, which can be recruited to participate in immune responses against novel, antigenically related pathogens.

Technological progress is pivotal in unraveling the complexity of cross-reactive Trm cells. High-parameter imaging modalities such as Imaging Mass Cytometry (IMC) or Multiplexed Ion Beam Imaging (MIBI), provide detailed spatial maps of Trm cells within their tissue context, revealing their interactions with other immune and stromal cells [111, 112]. Furthermore, the integration of lineage tracing with single-cell TCR sequencing have been used in mice to reconstruct clonal histories, tracing the fate of individual T cell clones across differentiation states and anatomical sites [113, 114] A transformative approach for the de novo discovery of cross-reactive Trm cells involves the epigenetic definition of residency states. In our recent generation of a methylome atlas of memory T cells from six human somatic tissues, we established a core epigenetic signature that distinguishes bona fide Trm cells from circulating populations [70]. This resource enables a powerful discovery pipeline: by first epigenetically identifying the stable Trm cell compartment in a tissue, we can then probe these pre-positioned residents for unexpected antigen specificities. This strategy will be key to identifying pre-existing antigen–reactive CD4⁺ Trm in unexposed individuals, revealing that tissue as a reservoir for cross-reactive clones against pathogens never encountered.

Collectively, these advanced methodologies, from high-dimensional imaging and clonal tracking to epigenetic cartography, are shifting the paradigm from simply observing cross-reactivity to systematically discovering the full scope of the cross-reactive Trm cell repertoire across the human body. A summary of Trm locations together with the discovery methods used to study them is provided in Table 1.

## Benefits and risks of cross reactive Trm cells in clinics

The ability of cross-reactive Trm cells to provide broad, frontline immunity positions them as highly attractive targets for clinical intervention. This potential is being explored across infectious disease and oncology. In vaccinology, platforms designed to generate lung-resident Trm cells are a promising strategy against viral variants. For instance, the OVX836 heptameric nucleoprotein vaccine induces cross-reactive CD8^+^ Trm cells in the lungs, conferring heterologous protection against diverse influenza strains [115, 116]. Similarly, pre-existing cross-reactive Trm cells, likely induced by common cold coronavirus (e.g., HCoV-OC43) infections, have been implicated in mitigating COVID-19 severity [117], highlighting the natural clinical benefit of this phenomenon. Beside viruses, Trm cells recognizing conserved bacterial antigens, such as *Mycobacterium tuberculosis* ESAT-6 can enhance protection against related bacterial strains [118]. In oncology, the therapeutic value of Trm cells is underscored by their correlation with improved survival in multiple cancers. Tumor-infiltrating Trm can target shared tumor-associated antigens (TAAs) and neoantigens, mediating sustained cross-protective immunity against cancers with high mutational burdens [91, 119, 120].

However, the potent and persistent nature of Trm cells constitutes a double-edged sword. The same mechanisms that enable durable protection can also drive pathology when Trm cross-react with self-antigens. Overactive or dysregulated Trm cell responses are increasingly implicated in the chronic inflammation that characterizes autoimmunity and inflammatory conditions such as psoriasis and inflammatory bowel disease [54, 121]. Therefore, a central challenge in harnessing Trm cells is to maximize their broad protective capacity while minimizing off-target autoimmunity. The risk-benefit calculus necessitates the development of sophisticated control strategies. Future therapeutic approaches may include: fine-tuning Trm cells function by epigenetic and metabolic reprogramming of Trm cells activity without eliminating this crucial population [107, 122], selectively depleting autoreactive Trm cell populations using engineered CAR-T cell therapy [123], and knocking out Trm cell-specific transcription factors like Runx3 to impair their tissue residency using CRISPR-based gene editing techniques [123, 124].

## Challenges and future directions

Despite advances in Trm cell biology, technical and therapeutic challenges remain. Human tissue viability is limited, and current animal models may not fully recapitulate Trm cells diversity and function, highlighting the need for high-dimensional profiling, longitudinal tracking, and single-cell TCR repertoire analysis to map cross-reactive Trm cell populations. Therapeutically, vaccination and adoptive transfer of engineered Trm cells with defined specificities offer promise for infection and cancer, while Trm cell inhibition may contribute to the modulation of autoimmune responses. Early clinical studies have explored cross-reactive Trm cells in skin disorders and vaccination, but broader trials are needed to fully explore their protective and pathological potential.

## Conclusions

As a critical cornerstone of adaptive immunity, the functional potency of Trm cells is profoundly amplified by the inherent property of TCR cross-reactivity, which enables a limited TCR repertoire to recognize a diverse array of antigenic threats. In this review, we have highlighted that this cross-reactivity endows Trm cell populations with a dual nature: they are indispensable sentinels providing broad, tissue-tropic protection against pathogens and tumors, yet they also carry the latent risk of exacerbating autoimmune pathology when recognition goes awry. We also discuss the central challenge and opportunity lie in the therapeutic modulation of cross-reactive Trm cells. Emerging strategies aim to strategically harness this property, perhaps through targeted vaccination or adoptive cell transfer, to bolster immune surveillance in mucosal tissues and solid tumors. Conversely, developing precise methods to restrain or delete pathogenic cross-reactive Trm clones without compromising overall tissue immunity offers promising avenues for treating autoimmune and chronic inflammatory diseases. Ultimately, decoding the precise rules governing Trm cell cross-reactivity will be key to leveraging these powerful cells, transforming them from immunological study into potent next-generation immunotherapies.

## Electronic Supplementary Material

Below is the link to the electronic supplementary material.

Supplementary new reference list

## Data Availability

Not applicable.

## Abbreviations

allo-HCT: Allogeneic hematopoietic stem cell transplant
CDR: Complementarity determining regions
CMV: Cytomegalovirus
CNS: Central nervous system
EAE: Experimental autoimmune encephalomyelitis
EBV: Epstein-Barr virus
GvHD: Graft-versus-host disease
HCC: Hepatocellular carcinoma
ICI: Immune checkpoint inhibitors
IMC: IMAGING Mass Cytometry
MHC: Major Histocompatibility Complex
MIBI: Multiplexed Ion Beam Imaging
MMR: Measles, mumps, rubella
NP: Nucleoprotein
TAAs: Tumor-associated antigens
TAM: Tumor-associated macrophages
Tcm: Central memory T cells
TCR: T cell receptor
Tem: Effector memory T cells
TME: Tumor microenvironment
Trm: Tissue-resident memory T cells

## References

[1] Sallusto F, Lenig D, Förster R, Lipp M, Lanzavecchia A. Two subsets of memory T lymphocytes with distinct homing potentials and effector functions. Nature. 1999;401(6754):708–12. 10537110 10.1038/44385

[2] Radbruch A, McGrath MA, Siracusa F, Hoffmann U, Sercan-Alp Ö, Hutloff A, et al. Homeostasis and durability of T-cell memory-the resting and the restless t-cell memory. Cold Spring Harb Perspect Biol. 2021;13(7). 10.1101/cshperspect.a038083 PMC8247562 33903153

[3] Shoeran G, Anand N. Interplay of autophagy and Th1/Th2-mediated macrophage polarization in host-pathogen dynamics. Front Cell Infect Microbiol. 2025;15:1679514. 41112570 10.3389/fcimb.2025.1679514 PMC12528116

[4] Schirrmacher V. Bone Marrow: The Central Immune System. Immuno. 2023;3(3):289–329.

[5] Reina-Campos M, Monell A, Ferry A, Luna V, Cheung KP, Galletti G, et al. Tissue-resident memory CD8 T cell diversity is spatiotemporally imprinted. Nature. 2025;639(8054):483–92. 39843748 10.1038/s41586-024-08466-x PMC11903307

[6] Behr FM, Parga-Vidal L, Kragten NAM, van Dam TJP, Wesselink TH, et al. Tissue-resident memory CD8(+) T cells shape local and systemic secondary T cell responses. Nat Immunol. 2020;21(9):1070–81. 32661361 10.1038/s41590-020-0723-4

[7] Fonseca R, Beura LK, Quarnstrom CF, Ghoneim HE, Fan Y, Zebley CC, Scott MC, Fares-Frederickson NJ, Wijeyesinghe S, Thompson EA, et al. Developmental plasticity allows outside-in immune responses by resident memory T cells. Nat Immunol. 2020;21(4):412–21. 32066954 10.1038/s41590-020-0607-7 PMC7096285

[8] Cendón C, Du W, Durek P, Liu YC, Alexander T, Serene L, et al. Resident memory CD4(+) T lymphocytes mobilize from bone marrow to contribute to a systemic secondary immune reaction. Eur J Immunol. 2022;52(5):737–52. 35245389 10.1002/eji.202149726

[9] Koda Y, Teratani T, Chu PS, Hagihara Y, Mikami Y, Harada Y, et al. CD8(+) tissue-resident memory T cells promote liver fibrosis resolution by inducing apoptosis of hepatic stellate cells. Nat Commun. 2021;12(1):4474. 34294714 10.1038/s41467-021-24734-0 PMC8298513

[10] Huang B, Lyu Z, Qian Q, Chen Y, Zhang J, Li B, et al. NUDT1 promotes the accumulation and longevity of CD103(+) T(RM) cells in primary biliary cholangitis. J Hepatol. 2022;77(5):1311–24. 35753523 10.1016/j.jhep.2022.06.014

[11] Ostkamp P, Deffner M, Schulte-Mecklenbeck A, Wünsch C, Lu IN, Wu GF, et al. A single-cell analysis framework allows for characterization of CSF leukocytes and their tissue of origin in multiple sclerosis. Sci Transl Med. 2022;14(673):eadc9778. 36449599 10.1126/scitranslmed.adc9778

[12] Cheuk S, Schlums H, Gallais Sérézal I, Martini E, Chiang SC, Marquardt N, et al. CD49a Expression Defines Tissue-Resident CD8(+) T Cells Poised for Cytotoxic Function in Human Skin. Immunity. 2017;46(2):287–300. 28214226 10.1016/j.immuni.2017.01.009 PMC5337619

[13] Zitti B, Hoffer E, Zheng W, Pandey RV, Schlums H, Perinetti Casoni G, et al. Human skin-resident CD8(+) T cells require RUNX2 and RUNX3 for induction of cytotoxicity and expression of the integrin CD49a. Immunity. 2023;56(6):1285–e13021287. 37269830 10.1016/j.immuni.2023.05.003

[14] Schøller AS, Nazerai L, Christensen JP, Thomsen AR. Functionally Competent, PD-1(+) CD8(+) Trm Cells Populate the Brain Following Local Antigen Encounter. Front Immunol. 2020;11:595707. 33603737 10.3389/fimmu.2020.595707 PMC7884456

[15] Steinert EM, Schenkel JM, Fraser KA, Beura LK, Manlove LS, Igyártó BZ, et al. Quantifying Memory CD8 T Cells Reveals Regionalization of Immunosurveillance. Cell. 2015;161(4):737–49. 25957682 10.1016/j.cell.2015.03.031 PMC4426972

[16] Schneider Revueltas E, Ferreira-Gomes M, Guerra GM, Durek P, Heinrich F, Casanovas Subirana A, et al. Surface CD69-Negative CD4 and CD8 Bone Marrow-Resident Human Memory T Cells. Eur J Immunol. 2025;55(5):e202451529. 40375826 10.1002/eji.202451529 PMC12082384

[17] Okhrimenko A, Grun JR, Westendorf K, Fang Z, Reinke S, von Roth P, et al. Human memory T cells from the bone marrow are resting and maintain long-lasting systemic memory. Proc Natl Acad Sci U S A. 2014;111(25):9229–34. 24927527 10.1073/pnas.1318731111 PMC4078840

[18] Sercan Alp Ö, Durlanik S, Schulz D, McGrath M, Grün JR, Bardua M, Ikuta K, et al. Memory CD8 + T cells colocalize with IL-7 + stromal cells in bone marrow and rest in terms of proliferation and transcription. Eur J Immunol. 2015;45(4):975–87. 25639669 10.1002/eji.201445295 PMC4415462

[19] Chang HD, Tokoyoda K, Radbruch A. Immunological memories of the bone marrow. Immunol Rev. 2018;283(1):86–98. 29664564 10.1111/imr.12656 PMC5947123

[20] Siracusa F, Alp OS, Maschmeyer P, McGrath M, Mashreghi MF, Hojyo S, Chang HD, Tokoyoda K, Radbruch A. Maintenance of CD8(+) memory T lymphocytes in the spleen but not in the bone marrow is dependent on proliferation. Eur J Immunol. 2017;47(11):1900–5. 28815584 10.1002/eji.201747063 PMC5698754

[21] Tokoyoda K, Zehentmeier S, Hegazy AN, Albrecht I, Grun JR, Lohning M, Radbruch A. Professional memory CD4 + T lymphocytes preferentially reside and rest in the bone marrow. Immunity. 2009;30(5):721–30. 19427242 10.1016/j.immuni.2009.03.015

[22] Kurd NS, He Z, Louis TL, Milner JJ, Omilusik KD, Jin W, et al. Early precursors and molecular determinants of tissue-resident memory CD8(+) T lymphocytes revealed by single-cell RNA sequencing. Sci Immunol. 2020;5(47). 10.1126/sciimmunol.aaz6894 PMC7341730 32414833

[23] Kumar BV, Ma W, Miron M, Granot T, Guyer RS, Carpenter DJ, et al. Human Tissue-Resident Memory T Cells Are Defined by Core Transcriptional and Functional Signatures in Lymphoid and Mucosal Sites. Cell Rep. 2017;20(12):2921–34. 28930685 10.1016/j.celrep.2017.08.078 PMC5646692

[24] Sewell AK. Why must T cells be cross-reactive? Nat Rev Immunol. 2012;12(9):669–77. 22918468 10.1038/nri3279 PMC7097784

[25] Koutsakos M, Illing PT, Nguyen THO, Mifsud NA, Crawford JC, Rizzetto S, et al. Human CD8(+) T cell cross-reactivity across influenza A, B and C viruses. Nat Immunol. 2019;20(5):613–25. 30778243 10.1038/s41590-019-0320-6

[26] Zheng MZM, Tan TK, Villalon-Letelier F, Lau H, Deng YM, Fritzlar S, et al. Single-cycle influenza virus vaccine generates lung CD8(+) Trm that cross-react against viral variants and subvert virus escape mutants. Sci Adv. 2023;9(36):eadg3469. 37683004 10.1126/sciadv.adg3469 PMC10491285

[27] Ahmed R, Gray D. Immunological memory and protective immunity: understanding their relation. Science. 1996;272(5258):54–60. 8600537 10.1126/science.272.5258.54

[28] McGregor DD, Gowans JL, Survival of homografts of skin in rats depleted of lymphocytes by chronic drainage from the thoracic duct. Lancet. 1964;1(7334):629–32. 14107928 10.1016/s0140-6736(64)91451-5

[29] Teijaro JR, Turner D, Pham Q, Wherry EJ, Lefrançois L, Farber DL. Cutting edge: Tissue-retentive lung memory CD4 T cells mediate optimal protection to respiratory virus infection. J Immunol. 2011;187(11):5510–4. 22058417 10.4049/jimmunol.1102243 PMC3221837

[30] Mackay LK, Kallies A. Transcriptional Regulation of Tissue-Resident Lymphocytes. Trends Immunol. 2017;38(2):94–103. 27939451 10.1016/j.it.2016.11.004

[31] Clark RA, Watanabe R, Teague JE, Schlapbach C, Tawa MC, Adams N, et al. Skin effector memory T cells do not recirculate and provide immune protection in alemtuzumab-treated CTCL patients. Sci Transl Med. 2012;4(117):117ra117. 10.1126/scitranslmed.3003008 PMC3373186 22261031

[32] Zhang P, Miao J, Yu H, Yu H, Liu C, Zhao L, et al. Sympathetic-epithelial crosstalk governs tissue-resident memory T cell immunosurveillance in the skin. Cell. 2026. 10.1016/j.cell.2025.12.043 41616781

[33] Hullegie-Peelen DM, Tejeda Mora H, Hesselink DA, Bindels EM, van den Bosch TP, Clahsen-van Groningen MC, et al. Virus-specific TRM cells of both donor and recipient origin reside in human kidney transplants. JCI Insight. 2023;8(21). 10.1172/jci.insight.172681 PMC10721264 37751288

[34] Kalinoski H, Daoud A, Rusinkevich V, Jurčová I, Talor MV, Welsh RA, et al. Injury-induced myosin-specific tissue-resident memory T cells drive immune checkpoint inhibitor myocarditis. Proc Natl Acad Sci U S A. 2024;121(42):e2323052121. 39378095 10.1073/pnas.2323052121 PMC11494310

[35] de Jong MJM, Depuydt MAC, Schaftenaar FH, Liu K, Maters D, Wezel A, et al. Resident Memory T Cells in the Atherosclerotic Lesion Associate With Reduced Macrophage Content and Increased Lesion Stability. Arterioscler Thromb Vasc Biol. 2024;44(6):1318–29. 38634281 10.1161/ATVBAHA.123.320511

[36] Ganley M, Holz LE, Minnell JJ, de Menezes MN, Burn OK, Poa KCY, et al. mRNA vaccine against malaria tailored for liver-resident memory T cells. Nat Immunol. 2023;24(9):1487–98. 37474653 10.1038/s41590-023-01562-6

[37] Kirchmeier D, Deng Y, Rieble L, Böni M, Läderach F, Schuhmachers P, et al. Epstein-Barr virus infection induces tissue-resident memory T cells in mucosal lymphoid tissues. JCI Insight 2024;9(20). 10.1172/jci.insight.173489 PMC11530129 39264727

[38] Lin YH, Duong HG, Limary AE, Kim ES, Hsu P, Patel SA, et al. Small intestine and colon tissue-resident memory CD8(+) T cells exhibit molecular heterogeneity and differential dependence on Eomes. Immunity. 2023;56(1):207–e223208. 36580919 10.1016/j.immuni.2022.12.007 PMC9904390

[39] van de Wall S, Anthony SM, Hancox LS, Pewe LL, Langlois RA, Zehn D, et al. Dynamic landscapes and protective immunity coordinated by influenza-specific lung-resident memory CD8(+) T cells revealed by intravital imaging. Immunity. 2024;57(8):1878–e18921875. 39043185 10.1016/j.immuni.2024.06.016 PMC12001675

[40] Peng T, Phasouk K, Bossard E, Klock A, Jin L, Laing KJ, et al. Distinct populations of antigen-specific tissue-resident CD8 + T cells in human cervix mucosa. JCI Insight. 2021;6(15). 10.1172/jci.insight.149950 PMC8410090 34156975

[41] Hobson R, Levy SHS, Singal CMS, Flaherty D, Xiao H, Ciener B, et al. Clonal CD8(+) T cells populate the leptomeninges and coordinate with immune cells in human degenerative brain diseases. Nat Immunol. 2026;27(2):323–35. 41593242 10.1038/s41590-025-02401-6 PMC12864034

[42] Liu Y, Wang H, Taylor M, Cook C, Martínez-Berdeja A, North JP, et al. Classification of human chronic inflammatory skin disease based on single-cell immune profiling. Sci Immunol. 2022;7(70):eabl9165. 35427179 10.1126/sciimmunol.abl9165 PMC9301819

[43] Boyman O, et al. Spontaneous development of psoriasis in a new animal model shows an essential role for resident T cells and tumor necrosis factor-alpha. J Exp Med. 2004 Mar 1;199(5):731-41 10.1084/jem.20031482 10.1084/jem.20031482 PMC2213300 14981113

[44] Clark RA, Chong B, Mirchandani N, Brinster NK, Yamanaka K, Dowgiert RK, et al. The vast majority of CLA+ T cells are resident in normal skin. J Immunol. 2006 Apr 1;176(7):4431–9. 10.4049/jimmunol.176.7.4431 10.4049/jimmunol.176.7.4431 16547281

[45] Mizukawa Y, Shiohara T. Trauma-localized fixed drug eruption: involvement of burn scars, insect bites and venipuncture sites. Dermatology. 2002;205(2):159–61. 12218232 10.1159/000063892

[46] Shiohara T, Ushigome Y, Kano Y, Takahashi R. Crucial Role of Viral Reactivation in the Development of Severe Drug Eruptions: a Comprehensive Review. Clin Rev Allergy Immunol. 2015;49(2):192–202. 24736996 10.1007/s12016-014-8421-3

[47] Siracusa F, McGrath MA, Maschmeyer P, et al. Nonfollicular reactivation of bone marrow resident memory CD4 T cells in immune clusters of the bone marrow. Proc Natl Acad Sci U S A. 2018 Feb 6;115(6):1334–9. 10.1073/pnas.1715618115 10.1073/pnas.1715618115 PMC5819416 29358404

[48] Li J, Reinke S, Shen Y, Schollmeyer L, Liu YC, Wang Z, et al. A ubiquitous bone marrow reservoir of preexisting SARS-CoV-2-reactive memory CD4(+) T lymphocytes in unexposed individuals. Front Immunol. 2022;13:1004656. 36268016 10.3389/fimmu.2022.1004656 PMC9576920

[49] Nelson CE, Foreman TW, Fukutani ER, Kauffman KD, Sakai S, Fleegle JD, et al. IL-10 suppresses T cell expansion while promoting tissue-resident memory cell formation during SARS-CoV-2 infection in rhesus macaques. PLoS Pathog. 2024;20(7):e1012339. 38950078 10.1371/journal.ppat.1012339 PMC11244803

[50] Siracusa F, Durek P, McGrath MA, Sercan-Alp Ö, Rao A, Du W, et al. CD69(+) memory T lymphocytes of the bone marrow and spleen express the signature transcripts of tissue-resident memory T lymphocytes. Eur J Immunol. 2019;49(6):966–8. 30673129 10.1002/eji.201847982 PMC6563480

[51] Luo S, Zhang P, Wang Y, Huang Y, Ma X, Deng Q, et al. Adenoviruses vectored hepatitis C virus vaccine cocktails induce broadly specific immune responses against multi-genotypic HCV in mice. Biomed Pharmacother. 2024;170:115901. 38056238 10.1016/j.biopha.2023.115901

[52] Iwanaga N, Chen K, Yang H, Lu S, Hoffmann JP, Wanek A, et al. Vaccine-driven lung TRM cells provide immunity against Klebsiella via fibroblast IL-17R signaling. Sci Immunol. 2021;6(63):eabf1198. 34516780 10.1126/sciimmunol.abf1198 PMC8796208

[53] Jiao W, Long KD, Young T, Muntnich CB, Rey AP, Khwajazadah M, et al. Immunogenomic landscape of T cell repertoire after human lung transplantation and its clinical significance. *medRxiv* 2025. 10.1016/j.ajt.2026.06.009 PMC13403488 42309217

[54] Yokoi T, Murakami M, Kihara T, Seno S, Arase M, Wing JB, et al. Identification of a unique subset of tissue-resident memory CD4(+) T cells in Crohn’s disease. Proc Natl Acad Sci U S A. 2023;120(1):e2204269120. 36574662 10.1073/pnas.2204269120 PMC9910620

[55] Jiao W, Martinez M, Muntnich CB, Zuber J, Parks C, Obradovic A, et al. Dynamic establishment of recipient resident memory T cell repertoire after human intestinal transplantation. EBioMedicine. 2024;101:105028. 38422982 10.1016/j.ebiom.2024.105028 PMC10944178

[56] Dravid P, Murthy S, Attia Z, Cassady C, Chandra R, Trivedi S, et al. Phenotype and fate of liver-resident CD8 T cells during acute and chronic hepacivirus infection. PLoS Pathog. 2023;19(10):e1011697. 37812637 10.1371/journal.ppat.1011697 PMC10602381

[57] Zimmer CL, von Seth E, Buggert M, Strauss O, Hertwig L, Nguyen S, et al. A biliary immune landscape map of primary sclerosing cholangitis reveals a dominant network of neutrophils and tissue-resident T cells. Sci Transl Med. 2021;13(599). 10.1126/scitranslmed.abb3107 34162753

[58] Abou-Daya KI, Tieu R, Zhao D, Rammal R, Sacirbegovic F, Williams AL, et al. Resident memory T cells form during persistent antigen exposure leading to allograft rejection. Sci Immunol. 2021;6(57). 10.1126/sciimmunol.abc8122 PMC8103522 33741656

[59] Schøller AS, Fonnes M, Nazerai L, Christensen JP, Thomsen AR. Local Antigen Encounter Is Essential for Establishing Persistent CD8(+) T-Cell Memory in the CNS. Front Immunol. 2019;10:351. 30886617 10.3389/fimmu.2019.00351 PMC6409353

[60] Altendorfer B, Unger MS, Poupardin R, Hoog A, Asslaber D, Gratz IK, et al. Transcriptomic Profiling Identifies CD8(+) T Cells in the Brain of Aged and Alzheimer’s Disease Transgenic Mice as Tissue-Resident Memory T Cells. J Immunol. 2022;209(7):1272–85. 36165202 10.4049/jimmunol.2100737 PMC9515311

[61] Mackay LK, Rahimpour A, Ma JZ, Collins N, Stock AT, Hafon ML, et al. The developmental pathway for CD103(+)CD8 + tissue-resident memory T cells of skin. Nat Immunol. 2013;14(12):1294–301. 24162776 10.1038/ni.2744

[62] Mackay LK, Wynne-Jones E, Freestone D, Pellicci DG, Mielke LA, Newman DM, et al. T-box Transcription Factors Combine with the Cytokines TGF-β and IL-15 to Control Tissue-Resident Memory T Cell Fate. Immunity. 2015;43(6):1101–11. 26682984 10.1016/j.immuni.2015.11.008

[63] Wein AN, McMaster SR, Takamura S, Dunbar PR, Cartwright EK, Hayward SL, et al. CXCR6 regulates localization of tissue-resident memory CD8 T cells to the airways. J Exp Med. 2019;216(12):2748–62. 31558615 10.1084/jem.20181308 PMC6888981

[64] Tkachev V, Kaminski J, Potter EL, Furlan SN, Yu A, Hunt DJ, et al. Spatiotemporal single-cell profiling reveals that invasive and tissue-resident memory donor CD8(+) T cells drive gastrointestinal acute graft-versus-host disease. Sci Transl Med. 2021;13(576). 10.1126/scitranslmed.abc0227 PMC9469805 33441422

[65] Ozga AJ, Chow MT, Lopes ME, Servis RL, Di Pilato M, Dehio P, et al. CXCL10 chemokine regulates heterogeneity of the CD8(+) T cell response and viral set point during chronic infection. Immunity. 2022;55(1):82–e9788. 34847356 10.1016/j.immuni.2021.11.002 PMC8755631

[66] Park CO, Fu X, Jiang X, Pan Y, Teague JE, Collins N, et al. Staged development of long-lived T-cell receptor αβ T(H)17 resident memory T-cell population to Candida albicans after skin infection. J Allergy Clin Immunol. 2018;142(2):647–62. 29128674 10.1016/j.jaci.2017.09.042 PMC5943196

[67] Herrera-De La Mata S, Ramírez-Suástegui C, Mistry H, Castañeda-Castro FE, Kyyaly MA, Simon H, et al. Cytotoxic CD4(+) tissue-resident memory T cells are associated with asthma severity. Med. 2023;4(12):875–e897878. 37865091 10.1016/j.medj.2023.09.003 PMC10964988

[68] Lutshumba J, Ochiai E, Sa Q, Anand N, Suzuki Y. Selective upregulation of transcripts for six molecules related to T Cell costimulation and phagocyte recruitment and activation among 734 immunity-related genes in the brain during perforin-dependent, CD8(+) T Cell-mediated elimination of toxoplasma gondii Cysts. mSystems. 2020;5(2). 10.1128/mSystems.00189-20 PMC7159899 32291349

[69] Dong J, Chang H-D, Radbruch A. Epigenetic imprinting of immunological memory. In: *Epigenetics - *A Different Way of Looking at Genetics*.* Edited by Doerfler W, Böhm P. Cham: Springer International Publishing; 2016:53–67.

[70] Deng X, Du W, Gasparoni G, Salhab A, Nordström K, Li J, et al. Methylomes of human CD4 and CD8 memory T lymphocytes reveal tissue-specific epigenetic signatures for maintenance and recall function. Immun Inflamm. 2025;1(1):13. 41262706 10.1007/s44466-025-00009-x PMC12623507

[71] Tsuchiya Y, Namiuchi Y, Wako H, Tsurui H. A study of CDR3 loop dynamics reveals distinct mechanisms of peptide recognition by T-cell receptors exhibiting different levels of cross-reactivity. Immunology. 2018;153(4):466–78. 28992359 10.1111/imm.12849 PMC5838422

[72] Cole DK, Miles KM, Madura F, Holland CJ, Schauenburg AJ, Godkin AJ, et al. T-cell receptor (TCR)-peptide specificity overrides affinity-enhancing TCR-major histocompatibility complex interactions. J Biol Chem. 2014;289(2):628–38. 24196962 10.1074/jbc.M113.522110 PMC3887192

[73] Gleimer M, Wahl AR, Hickman HD, Abi-Rached L, Norman PJ, Guethlein LA, et al. Although divergent in residues of the peptide binding site, conserved chimpanzee Patr-AL and polymorphic human HLA-A*02 have overlapping peptide-binding repertoires. J Immunol. 2011;186(3):1575–88. 21209280 10.4049/jimmunol.1002990 PMC3124313

[74] Hamza H, Ghosh M, Löffler MW, Rammensee HG, Planz O. Identification and relative abundance of naturally presented and cross-reactive influenza A virus MHC class I-restricted T cell epitopes. Emerg Microbes Infect. 2024;13(1):2306959. 38240239 10.1080/22221751.2024.2306959 PMC10854457

[75] Holland CJ, Rizkallah PJ, Vollers S, Calvo-Calle JM, Madura F, Fuller A, Sewell AK, Stern LJ, Godkin A, Cole DK. Minimal conformational plasticity enables TCR cross-reactivity to different MHC class II heterodimers. Sci Rep. 2012;2:629. 22953050 10.1038/srep00629 PMC3432979

[76] Welsh RM, Selin LK. No one is naive: the significance of heterologous T-cell immunity. Nat Rev Immunol. 2002;2(6):417–26. 12093008 10.1038/nri820

[77] Su LF, Kidd BA, Han A, Kotzin JJ, Davis MM. Virus-specificCD4(+) memory-phenotype T cells are abundant in unexposed adults. Immunity 2013, 38(2):373–383. 10.1016/j.immuni.2012.10.021 PMC3626102 23395677

[78] Chaisawangwong W, Wang H, Kouo T, Salathe SF, Isser A, Bieler JG, et al. Cross-reactivity of SARS-CoV-2- and influenza A-specific T cells in individuals exposed to SARS-CoV-2. JCI Insight. 2022;7(18). 10.1172/jci.insight.158308 PMC9675569 36134660

[79] Grifoni A, Weiskopf D, Ramirez SI, Mateus J, Dan JM, Moderbacher CR, et al. Targets of T Cell Responses to SARS-CoV-2 Coronavirus in Humans with COVID-19 Disease and Unexposed Individuals. Cell. 2020;181(7):1489–e15011415. 32473127 10.1016/j.cell.2020.05.015 PMC7237901

[80] Mateus J, Grifoni A, Tarke A, Sidney J, Ramirez SI, Dan JM, et al. Selective and cross-reactive SARS-CoV-2 T cell epitopes in unexposed humans. Science. 2020;370(6512):89–94. 32753554 10.1126/science.abd3871 PMC7574914

[81] Pothast CR, Dijkland RC, Thaler M, Hagedoorn RS, Kester MGD, Wouters AK, et al. SARS-CoV-2-specific CD4(+) and CD8(+) T cell responses can originate from cross-reactive CMV-specific T cells. Elife. 2022;11. 10.7554/eLife.82050 PMC9822249 36408799

[82] Keeton R, Tincho MB, Ngomti A, Baguma R, Benede N, Suzuki A, et al. T cell responses to SARS-CoV-2 spike cross-recognize Omicron. Nature. 2022;603(7901):488–92. 35102311 10.1038/s41586-022-04460-3 PMC8930768

[83] Xiang SD, Gao Q, Wilson KL, Heyerick A, Plebanski M. A Nanoparticle Based Sp17 Peptide Vaccine Exposes New Immuno-Dominant and Species Cross-reactive B Cell Epitopes. Vaccines (Basel). 2015;3(4):875–93. 26529027 10.3390/vaccines3040875 PMC4693223

[84] Kover K, Hegre O, Popiela H, Biggs T, Moore WV. Cross-reactivity of organs in allograft rejection. Comparison of effect of thyroid allografts on established islet allografts. Diabetes. 1987;36(11):1268–70. 3117605 10.2337/diab.36.11.1268

[85] Peereboom ETM, Matern BM, Tomosugi T, Niemann M, Drylewicz J, Joosten I, et al. T-Cell Epitopes Shared Between Immunizing HLA and Donor HLA Associate With Graft Failure After Kidney Transplantation. Front Immunol. 2021;12:784040. 34868064 10.3389/fimmu.2021.784040 PMC8637278

[86] Picarda E, Bézie S, Usero L, Ossart J, Besnard M, Halim H, et al. Cross-Reactive Donor-Specific CD8(+) Tregs Efficiently Prevent Transplant Rejection. Cell Rep. 2019;29(13):4245–e42554246. 31875536 10.1016/j.celrep.2019.11.106

[87] Wang J, Jelcic I, Mühlenbruch L, Haunerdinger V, Toussaint NC, Zhao Y, et al. HLA-DR15 Molecules Jointly Shape an Autoreactive T Cell Repertoire in Multiple Sclerosis. Cell. 2020;183(5):1264–e12811220. 33091337 10.1016/j.cell.2020.09.054 PMC7707104

[88] Reynolds CJ, Sim MJ, Quigley KJ, Altmann DM, Boyton RJ. Autoantigen cross-reactive environmental antigen can trigger multiple sclerosis-like disease. J Neuroinflammation. 2015;12:91. 25962509 10.1186/s12974-015-0313-9 PMC4432996

[89] Edwards J, Wilmott JS, Madore J, Gide TN, Quek C, Tasker A, et al. CD103(+) Tumor-Resident CD8(+) T Cells Are Associated with Improved Survival in Immunotherapy-Naïve Melanoma Patients and Expand Significantly During Anti-PD-1 Treatment. Clin Cancer Res. 2018;24(13):3036–45. 29599411 10.1158/1078-0432.CCR-17-2257

[90] Rosato PC, Wijeyesinghe S, Stolley JM, Nelson CE, Davis RL, Manlove LS, et al. Virus-specific memory T cells populate tumors and can be repurposed for tumor immunotherapy. Nat Commun. 2019;10(1):567. 30718505 10.1038/s41467-019-08534-1 PMC6362136

[91] Chiou SH, Tseng D, Reuben A, Mallajosyula V, Molina IS, Conley S, et al. Global analysis of shared T cell specificities in human non-small cell lung cancer enables HLA inference and antigen discovery. Immunity. 2021;54(3):586–e602588. 33691136 10.1016/j.immuni.2021.02.014 PMC7960510

[92] Simoni Y, Becht E, Fehlings M, Loh CY, Koo SL, Teng KWW, et al. Bystander CD8(+) T cells are abundant and phenotypically distinct in human tumour infiltrates. Nature. 2018;557(7706):575–9. 29769722 10.1038/s41586-018-0130-2

[93] Newman JH, Chesson CB, Herzog NL, Bommareddy PK, Aspromonte SM, Pepe R, Estupinian R, et al. Intratumoral injection of the seasonal flu shot converts immunologically cold tumors to hot and serves as an immunotherapy for cancer. Proc Natl Acad Sci U S A. 2020;117(2):1119–28. 31888983 10.1073/pnas.1904022116 PMC6969546

[94] Sefrin JP, Hillringhaus L, Mundigl O, Mann K, Ziegler-Landesberger D, Seul H, et al. Sensitization of Tumors for Attack by Virus-Specific CD8 + T-Cells Through Antibody-Mediated Delivery of Immunogenic T-Cell Epitopes. Front Immunol. 2019;10:1962. 31555260 10.3389/fimmu.2019.01962 PMC6712545

[95] Fusciello M, Ylösmäki E, Feola S, Uoti A, Martins B, Aalto K, et al. A novel cancer vaccine for melanoma based on an approved vaccine against measles, mumps, and rubella. Mol Ther Oncolytics. 2022;25:137–45. 35572195 10.1016/j.omto.2022.04.002 PMC9065466

[96] Zhang Y, Gabere M, Taylor MA, Simoes CC, Dumbauld C, Barro O, et al. Repurposing live attenuated trivalent MMR vaccine as cost-effective cancer immunotherapy. Front Oncol. 2022;12:1042250. 36457491 10.3389/fonc.2022.1042250 PMC9706410

[97] Ragone C, Manolio C, Cavalluzzo B, Mauriello A, Tornesello ML, Buonaguro FM, et al. Identification and validation of viral antigens sharing sequence and structural homology with tumor-associated antigens (TAAs). J Immunother Cancer. 2021;9(5). 10.1136/jitc-2021-002694 PMC8166618 34049932

[98] Quezada SA, Simpson TR, Peggs KS, Merghoub T, Vider J, Fan X, et al. Tumor-reactive CD4(+) T cells develop cytotoxic activity and eradicate large established melanoma after transfer into lymphopenic hosts. J Exp Med. 2010;207(3):637–50. 20156971 10.1084/jem.20091918 PMC2839156

[99] Oh DY, Kwek SS, Raju SS, Li T, McCarthy E, Chow E, et al. Intratumoral CD4(+) T Cells Mediate Anti-tumor Cytotoxicity in Human Bladder Cancer. Cell. 2020;181(7):1612–e16251613. 32497499 10.1016/j.cell.2020.05.017 PMC7321885

[100] Anand N, Peh KH, Kolesar JM. Macrophage repolarization as a therapeutic strategy for osteosarcoma. Int J Mol Sci. 2023;24(3). 10.3390/ijms24032858 PMC9917837 36769180

[101] Wu S, Anand N, Guo Z, Li M, Santiago Figueroa M, Jung L, et al. Bridging immune evasion and vascular dynamics for novel therapeutic frontiers in Hepatocellular Carcinoma. Cancers (Basel) 2025;17(11). 10.3390/cancers17111860 PMC12153674 40507341

[102] Ji Q, Perchellet A, Goverman JM. Viral infection triggers central nervous system autoimmunity via activation of CD8 + T cells expressing dual TCRs. Nat Immunol. 2010;11(7):628–34. 20526343 10.1038/ni.1888 PMC2900379

[103] Vanderlugt CL, Begolka WS, Neville KL, Katz-Levy Y, Howard LM, Eagar TN, et al. The functional significance of epitope spreading and its regulation by co-stimulatory molecules. Immunol Rev. 1998;164:63–72. 9795764 10.1111/j.1600-065x.1998.tb01208.x

[104] Vanderlugt CJ, Miller SD. Epitope spreading. Curr Opin Immunol. 1996;8(6):831–6. 8994863 10.1016/S0952-7915(96)80012-4 PMC7135770

[105] Pande H, Campo K, Shanley JD, Creeger ES, Artishevsky A, Gallez-Hawkins G, et al. Characterization of a 52K protein of murine cytomegalovirus and its immunological cross-reactivity with the DNA-binding protein ICP36 of human cytomegalovirus. J Gen Virol. 1991;72(Pt 6):1421–7. 1646282 10.1099/0022-1317-72-6-1421

[106] Chen YF, Hsieh AH, Wang LC, Yu KH, Kuo CF. Cytomegalovirus-Associated Autoantibody against TAF9 Protein in Patients with Systemic Lupus Erythematosus. J Clin Med. 2021;10(16). 10.3390/jcm10163722 PMC8396997 34442018

[107] Crowl JT, Heeg M, Ferry A, Milner JJ, Omilusik KD, Toma C, et al. Tissue-resident memory CD8(+) T cells possess unique transcriptional, epigenetic and functional adaptations to different tissue environments. Nat Immunol. 2022;23(7):1121–31. 35761084 10.1038/s41590-022-01229-8 PMC10041538

[108] Fernandez SA, Pelaez-Prestel HF, Fiyouzi T, Gomez-Perosanz M, Reiné J, Reche PA. Tetanus-diphtheria vaccine can prime SARS-CoV-2 cross-reactive T cells. Front Immunol. 2024;15:1425374. 39091504 10.3389/fimmu.2024.1425374 PMC11291333

[109] Hasan F, Chiu Y, Shaw RM, Wang J, Yee C. Hypoxia acts as an environmental cue for the human tissue-resident memory T cell differentiation program. JCI Insight. 2021;6(10). 10.1172/jci.insight.138970 PMC8262358 34027895

[110] Zhu HX, Yang SH, Gao CY, Bian ZH, Chen XM, Huang RR, et al. Targeting pathogenic CD8(+) tissue-resident T cells with chimeric antigen receptor therapy in murine autoimmune cholangitis. Nat Commun. 2024;15(1):2936. 38580644 10.1038/s41467-024-46654-5 PMC10997620

[111] Lutter L, Roosenboom B, Brand EC, Ter Linde JJ, Oldenburg B, van Lochem EG, et al. Homeostatic Function and Inflammatory Activation of Ileal CD8(+) Tissue-Resident T Cells Is Dependent on Mucosal Location. Cell Mol Gastroenterol Hepatol. 2021;12(5):1567–81. 34224909 10.1016/j.jcmgh.2021.06.022 PMC8551698

[112] Barsch M, Salié H, Schlaak AE, Zhang Z, Hess M, Mayer LS, et al. T-cell exhaustion and residency dynamics inform clinical outcomes in hepatocellular carcinoma. J Hepatol. 2022;77(2):397–409. 35367533 10.1016/j.jhep.2022.02.032

[113] Kok L, Dijkgraaf FE, Urbanus J, Bresser K, Vredevoogd DW, Cardoso RF, et al. A committed tissue-resident memory T cell precursor within the circulating CD8 + effector T cell pool. J Exp Med. 2020;217(10). 10.1084/jem.20191711 PMC7537386 32728699

[114] Tagkareli S, Salagianni M, Galani IE, Manioudaki M, Pavlos E, Thanopoulou K, et al. CD103 integrin identifies a high IL-10-producing FoxP3(+) regulatory T-cell population suppressing allergic airway inflammation. Allergy. 2022;77(4):1150–64. 34658046 10.1111/all.15144

[115] Del Campo J, Bouley J, Chevandier M, Rousset C, Haller M, Indalecio A, et al. OVX836 Heptameric Nucleoprotein Vaccine Generates Lung Tissue-Resident Memory CD8 + T-Cells for Cross-Protection Against Influenza. Front Immunol. 2021;12:678483. 34177921 10.3389/fimmu.2021.678483 PMC8223747

[116] Slütter B, Pewe LL, Lauer P, Harty JT. Cutting edge: rapid boosting of cross-reactive memory CD8 T cells broadens the protective capacity of the Flumist vaccine. J Immunol. 2013;190(8):3854–8. 23467935 10.4049/jimmunol.1202790 PMC3622175

[117] Hu C, Wang Z, Ren L, Hao Y, Zhu M, Jiang H, et al. Pre-existing anti-HCoV-OC43 immunity influences the durability and cross-reactivity of humoral response to SARS-CoV-2 vaccination. Front Cell Infect Microbiol. 2022;12:978440. 36118022 10.3389/fcimb.2022.978440 PMC9478943

[118] Woodworth JS, Clemmensen HS, Battey H, Dijkman K, Lindenstrøm T, Laureano RS, et al. A Mycobacterium tuberculosis-specific subunit vaccine that provides synergistic immunity upon co-administration with Bacillus Calmette-Guérin. Nat Commun. 2021;12(1):6658. 34795205 10.1038/s41467-021-26934-0 PMC8602668

[119] Fluckiger A, Daillère R, Sassi M, Sixt BS, Liu P, Loos F, et al. Cross-reactivity between tumor MHC class I-restricted antigens and an enterococcal bacteriophage. Science. 2020;369(6506):936–42. 32820119 10.1126/science.aax0701

[120] Helman SR, Stevanovic S, Campbell TE, Kwong MLM, Doran SL, Faquin WC, et al. Human Papillomavirus T-Cell Cross-reactivity in Cervical Cancer: Implications for Immunotherapy Clinical Trial Design. JAMA Netw Open. 2018;1(3):e180706. 30646017 10.1001/jamanetworkopen.2018.0706 PMC6324313

[121] Dong C, Lin L, Du J. Characteristics and sources of tissue-resident memory T cells in psoriasis relapse. Curr Res Immunol. 2023;4:100067. 37701270 10.1016/j.crimmu.2023.100067 PMC10493251

[122] Li C, Zhu B, Son YM, Wang Z, Jiang L, Xiang M, et al. The Transcription Factor Bhlhe40 Programs Mitochondrial Regulation of Resident CD8(+) T Cell Fitness and Functionality. Immunity. 2019;51(3):491–e507497. 31533057 10.1016/j.immuni.2019.08.013 PMC6903704

[123] Knudson CJ, Férez M, Alves-Peixoto P, Erkes DA, Melo-Silva CR, Tang L, et al. Mechanisms of Antiviral Cytotoxic CD4 T Cell Differentiation. J Virol. 2021;95(19):e0056621. 34260270 10.1128/JVI.00566-21 PMC8428409

[124] Hao Q, Kundu S, Shetty S, Tucker TA, Idell S, Tang H. Inducible general knockout of Runx3 profoundly reduces pulmonary cytotoxic CD8(+) T cells with minimal effect on outcomes in mice following influenza infection. Front Immunol. 2022;13:1011922. 36275778 10.3389/fimmu.2022.1011922 PMC9586250

[125] Anand N, Lutshumba J, Whitlow M, Abdelaziz MH, Mani R, Suzuki Y. Deficiency in indoleamine-2, 3-dioxygenase induces upregulation of guanylate binding protein 1 and inducible nitric oxide synthase expression in the brain during cerebral infection with Toxoplasma gondii in genetically resistant BALB/c mice but not in genetically susceptible C57BL/6 mice. Microbes Infect. 2022;24(3):104908. 34781010 10.1016/j.micinf.2021.104908 PMC9081123

