# Supplementary material for: Cross-reactive tissue-resident memory T lymphocytes—concepts, evidence, and open questions
Source: J Transl Med. 2026 May 7;24:659. doi: 10.1186/s12967-026-08137-7 (PMC13159280; doi:10.1186/s12967-026-08137-7)
Supplement: Supplementary file 1 — Supplementary new reference list [file 12967_2026_8137_MOESM1_ESM.docx]

**References**

1. Sallusto F, Lenig D, Förster R, Lipp M, Lanzavecchia A: **Two subsets of memory T lymphocytes with distinct homing potentials and effector functions**. *Nature* 1999, **401**(6754):708-712.

2. Radbruch A, McGrath MA, Siracusa F, Hoffmann U, Sercan-Alp Ö, Hutloff A, Tokoyoda K, Chang HD, Dong J: **Homeostasis and Durability of T-Cell Memory-The Resting and the Restless T-Cell Memory**. *Cold Spring Harb Perspect Biol* 2021, **13**(7).

3. Shoeran G, Anand N: **Interplay of autophagy and Th1/Th2-mediated macrophage polarization in host-pathogen dynamics**. *Front Cell Infect Microbiol* 2025, **15**:1679514.

4. Schirrmacher V: **Bone Marrow: The Central Immune System**. *Immuno* 2023, **3**(3):289-329.

5. Reina-Campos M, Monell A, Ferry A, Luna V, Cheung KP, Galletti G, Scharping NE, Takehara KK, Quon S, Challita PP *et al*: **Tissue-resident memory CD8 T cell diversity is spatiotemporally imprinted**. *Nature* 2025, **639**(8054):483-492.

6. Behr FM, Parga-Vidal L, Kragten NAM, van Dam TJP, Wesselink TH, Sheridan BS, Arens R, van Lier RAW, Stark R, van Gisbergen K: **Tissue-resident memory CD8(+) T cells shape local and systemic secondary T cell responses**. *Nat Immunol* 2020, **21**(9):1070-1081.

7. Fonseca R, Beura LK, Quarnstrom CF, Ghoneim HE, Fan Y, Zebley CC, Scott MC, Fares-Frederickson NJ, Wijeyesinghe S, Thompson EA *et al*: **Developmental plasticity allows outside-in immune responses by resident memory T cells**. *Nat Immunol* 2020, **21**(4):412-421.

8. Cendón C, Du W, Durek P, Liu YC, Alexander T, Serene L, Yang X, Gasparoni G, Salhab A, Nordström K *et al*: **Resident memory CD4(+) T lymphocytes mobilize from bone marrow to contribute to a systemic secondary immune reaction**. *Eur J Immunol* 2022, **52**(5):737-752.

9. Koda Y, Teratani T, Chu PS, Hagihara Y, Mikami Y, Harada Y, Tsujikawa H, Miyamoto K, Suzuki T, Taniki N *et al*: **CD8(+) tissue-resident memory T cells promote liver fibrosis resolution by inducing apoptosis of hepatic stellate cells**. *Nat Commun* 2021, **12**(1):4474.

10. Huang B, Lyu Z, Qian Q, Chen Y, Zhang J, Li B, Li Y, Liang J, Liu Q, Li Y *et al*: **NUDT1 promotes the accumulation and longevity of CD103(+) T(RM) cells in primary biliary cholangitis**. *J Hepatol* 2022, **77**(5):1311-1324.

11. Ostkamp P, Deffner M, Schulte-Mecklenbeck A, Wünsch C, Lu IN, Wu GF, Goelz S, De Jager PL, Kuhlmann T, Gross CC *et al*: **A single-cell analysis framework allows for characterization of CSF leukocytes and their tissue of origin in multiple sclerosis**. *Sci Transl Med* 2022, **14**(673):eadc9778.

12. Cheuk S, Schlums H, Gallais Sérézal I, Martini E, Chiang SC, Marquardt N, Gibbs A, Detlofsson E, Introini A, Forkel M *et al*: **CD49a Expression Defines Tissue-Resident CD8(+) T Cells Poised for Cytotoxic Function in Human Skin**. *Immunity* 2017, **46**(2):287-300.

13. Zitti B, Hoffer E, Zheng W, Pandey RV, Schlums H, Perinetti Casoni G, Fusi I, Nguyen L, Kärner J, Kokkinou E *et al*: **Human skin-resident CD8(+) T cells require RUNX2 and RUNX3 for induction of cytotoxicity and expression of the integrin CD49a**. *Immunity* 2023, **56**(6):1285-1302.e1287.

14. Schøller AS, Nazerai L, Christensen JP, Thomsen AR: **Functionally Competent, PD-1(+) CD8(+) Trm Cells Populate the Brain Following Local Antigen Encounter**. *Front Immunol* 2020, **11**:595707.

15. Steinert EM, Schenkel JM, Fraser KA, Beura LK, Manlove LS, Igyártó BZ, Southern PJ, Masopust D: **Quantifying Memory CD8 T Cells Reveals Regionalization of Immunosurveillance**. *Cell* 2015, **161**(4):737-749.

16. Schneider Revueltas E, Ferreira-Gomes M, Guerra GM, Durek P, Heinrich F, Casanovas Subirana A, Tokoyoda K, Dong J, Reinke S, Hardt S *et al*: **Surface CD69-Negative CD4 and CD8 Bone Marrow-Resident Human Memory T Cells**. *Eur J Immunol* 2025, **55**(5):e202451529.

17. Okhrimenko A, Grün JR, Westendorf K, Fang Z, Reinke S, von Roth P, Wassilew G, Kühl AA, Kudernatsch R, Demski S *et al*: **Human memory T cells from the bone marrow are resting and maintain long-lasting systemic memory**. *Proc Natl Acad Sci U S A* 2014, **111**(25):9229-9234.

18. Sercan Alp Ö, Durlanik S, Schulz D, McGrath M, Grün JR, Bardua M, Ikuta K, Sgouroudis E, Riedel R, Zehentmeier S *et al*: **Memory CD8+ T cells colocalize with IL-7+ stromal cells in bone marrow and rest in terms of proliferation and transcription**. *European Journal of Immunology* 2015, **45**(4):975-987.

19. Chang HD, Tokoyoda K, Radbruch A: **Immunological memories of the bone marrow**. *Immunol Rev* 2018, **283**(1):86-98.

20. Siracusa F, Alp OS, Maschmeyer P, McGrath M, Mashreghi MF, Hojyo S, Chang HD, Tokoyoda K, Radbruch A: **Maintenance of CD8(+) memory T lymphocytes in the spleen but not in the bone marrow is dependent on proliferation**. *Eur J Immunol* 2017, **47**(11):1900-1905.

21. Tokoyoda K, Zehentmeier S, Hegazy AN, Albrecht I, Grün JR, Löhning M, Radbruch A: **Professional memory CD4+ T lymphocytes preferentially reside and rest in the bone marrow**. *Immunity* 2009, **30**(5):721-730.

22. Kurd NS, He Z, Louis TL, Milner JJ, Omilusik KD, Jin W, Tsai MS, Widjaja CE, Kanbar JN, Olvera JG *et al*: **Early precursors and molecular determinants of tissue-resident memory CD8(+) T lymphocytes revealed by single-cell RNA sequencing**. *Sci Immunol* 2020, **5**(47).

23. Kumar BV, Ma W, Miron M, Granot T, Guyer RS, Carpenter DJ, Senda T, Sun X, Ho SH, Lerner H *et al*: **Human Tissue-Resident Memory T Cells Are Defined by Core Transcriptional and Functional Signatures in Lymphoid and Mucosal Sites**. *Cell Rep* 2017, **20**(12):2921-2934.

24. Sewell AK: **Why must T cells be cross-reactive?** *Nat Rev Immunol* 2012, **12**(9):669-677.

25. Koutsakos M, Illing PT, Nguyen THO, Mifsud NA, Crawford JC, Rizzetto S, Eltahla AA, Clemens EB, Sant S, Chua BY *et al*: **Human CD8(+) T cell cross-reactivity across influenza A, B and C viruses**. *Nat Immunol* 2019, **20**(5):613-625.

26. Zheng MZM, Tan TK, Villalon-Letelier F, Lau H, Deng YM, Fritzlar S, Valkenburg SA, Gu H, Poon LLM, Reading PC *et al*: **Single-cycle influenza virus vaccine generates lung CD8(+) Trm that cross-react against viral variants and subvert virus escape mutants**. *Sci Adv* 2023, **9**(36):eadg3469.

27. Ahmed R, Gray D: **Immunological memory and protective immunity: understanding their relation**. *Science* 1996, **272**(5258):54-60.

28. McGregor DD, Gowans JL: **SURVIVAL OF HOMOGRAFTS OF SKIN IN RATS DEPLETED OF LYMPHOCYTES BY CHRONIC DRAINAGE FROM THE THORACIC DUCT**. *Lancet* 1964, **1**(7334):629-632.

29. Teijaro JR, Turner D, Pham Q, Wherry EJ, Lefrançois L, Farber DL: **Cutting edge: Tissue-retentive lung memory CD4 T cells mediate optimal protection to respiratory virus infection**. *J Immunol* 2011, **187**(11):5510-5514.

30. Mackay LK, Kallies A: **Transcriptional Regulation of Tissue-Resident Lymphocytes**. *Trends in Immunology* 2017, **38**(2):94-103.

31. Clark RA, Watanabe R, Teague JE, Schlapbach C, Tawa MC, Adams N, Dorosario AA, Chaney KS, Cutler CS, Leboeuf NR *et al*: **Skin effector memory T cells do not recirculate and provide immune protection in alemtuzumab-treated CTCL patients**. *Sci Transl Med* 2012, **4**(117):117ra117.

32. Zhang P, Miao J, Yu H, Yu H, Liu C, Zhao L, Yang P, Zhou T, Zhang B: **Sympathetic-epithelial crosstalk governs tissue-resident memory T cell immunosurveillance in the skin**. *Cell* 2026.

33. Hullegie-Peelen DM, Tejeda Mora H, Hesselink DA, Bindels EM, van den Bosch TP, Clahsen-van Groningen MC, Dieterich M, Heidt S, Minnee RC, Verjans GM *et al*: **Virus-specific TRM cells of both donor and recipient origin reside in human kidney transplants**. *JCI Insight* 2023, **8**(21).

34. Kalinoski H, Daoud A, Rusinkevich V, Jurčová I, Talor MV, Welsh RA, Hughes D, Zemanová K, Stříž I, Hooper JE *et al*: **Injury-induced myosin-specific tissue-resident memory T cells drive immune checkpoint inhibitor myocarditis**. *Proc Natl Acad Sci U S A* 2024, **121**(42):e2323052121.

35. de Jong MJM, Depuydt MAC, Schaftenaar FH, Liu K, Maters D, Wezel A, Smeets HJ, Kuiper J, Bot I, van Gisbergen K *et al*: **Resident Memory T Cells in the Atherosclerotic Lesion Associate With Reduced Macrophage Content and Increased Lesion Stability**. *Arterioscler Thromb Vasc Biol* 2024, **44**(6):1318-1329.

36. Ganley M, Holz LE, Minnell JJ, de Menezes MN, Burn OK, Poa KCY, Draper SL, English K, Chan STS, Anderson RJ *et al*: **mRNA vaccine against malaria tailored for liver-resident memory T cells**. *Nat Immunol* 2023, **24**(9):1487-1498.

37. Kirchmeier D, Deng Y, Rieble L, Böni M, Läderach F, Schuhmachers P, Valencia-Camargo AD, Murer A, Caduff N, Chatterjee B *et al*: **Epstein-Barr virus infection induces tissue-resident memory T cells in mucosal lymphoid tissues**. *JCI Insight* 2024, **9**(20).

38. Lin YH, Duong HG, Limary AE, Kim ES, Hsu P, Patel SA, Wong WH, Indralingam CS, Liu YC, Yao P *et al*: **Small intestine and colon tissue-resident memory CD8(+) T cells exhibit molecular heterogeneity and differential dependence on Eomes**. *Immunity* 2023, **56**(1):207-223.e208.

39. Liu Y, Wang H, Taylor M, Cook C, Martínez-Berdeja A, North JP, Harirchian P, Hailer AA, Zhao Z, Ghadially R *et al*: **Classification of human chronic inflammatory skin disease based on single-cell immune profiling**. *Sci Immunol* 2022, **7**(70):eabl9165.

40. Iwanaga N, Chen K, Yang H, Lu S, Hoffmann JP, Wanek A, McCombs JE, Song K, Rangel-Moreno J, Norton EB *et al*: **Vaccine-driven lung TRM cells provide immunity against Klebsiella via fibroblast IL-17R signaling**. *Sci Immunol* 2021, **6**(63):eabf1198.

41. van de Wall S, Anthony SM, Hancox LS, Pewe LL, Langlois RA, Zehn D, Badovinac VP, Harty JT: **Dynamic landscapes and protective immunity coordinated by influenza-specific lung-resident memory CD8(+) T cells revealed by intravital imaging**. *Immunity* 2024, **57**(8):1878-1892.e1875.

42. Peng T, Phasouk K, Bossard E, Klock A, Jin L, Laing KJ, Johnston C, Williams NA, Czartoski JL, Varon D *et al*: **Distinct populations of antigen-specific tissue-resident CD8+ T cells in human cervix mucosa**. *JCI Insight* 2021, **6**(15).

43. Hobson R, Levy SHS, Singal CMS, Flaherty D, Xiao H, Ciener B, Reddy H, Zabinyakov N, Kim CY, Teich AF *et al*: **Clonal CD8(+) T cells populate the leptomeninges and coordinate with immune cells in human degenerative brain diseases**. *Nat Immunol* 2026, **27**(2):323-335.

44. Mackay LK, Rahimpour A, Ma JZ, Collins N, Stock AT, Hafon ML, Vega-Ramos J, Lauzurica P, Mueller SN, Stefanovic T *et al*: **The developmental pathway for CD103(+)CD8+ tissue-resident memory T cells of skin**. *Nat Immunol* 2013, **14**(12):1294-1301.

45. Mackay LK, Wynne-Jones E, Freestone D, Pellicci DG, Mielke LA, Newman DM, Braun A, Masson F, Kallies A, Belz GT *et al*: **T-box Transcription Factors Combine with the Cytokines TGF-β and IL-15 to Control Tissue-Resident Memory T Cell Fate**. *Immunity* 2015, **43**(6):1101-1111.

46. Wein AN, McMaster SR, Takamura S, Dunbar PR, Cartwright EK, Hayward SL, McManus DT, Shimaoka T, Ueha S, Tsukui T *et al*: **CXCR6 regulates localization of tissue-resident memory CD8 T cells to the airways**. *J Exp Med* 2019, **216**(12):2748-2762.

47. Siracusa F, Durek P, McGrath MA, Sercan-Alp Ö, Rao A, Du W, Cendón C, Chang HD, Heinz GA, Mashreghi MF *et al*: **CD69(+) memory T lymphocytes of the bone marrow and spleen express the signature transcripts of tissue-resident memory T lymphocytes**. *Eur J Immunol* 2019, **49**(6):966-968.

48. Tkachev V, Kaminski J, Potter EL, Furlan SN, Yu A, Hunt DJ, McGuckin C, Zheng H, Colonna L, Gerdemann U *et al*: **Spatiotemporal single-cell profiling reveals that invasive and tissue-resident memory donor CD8(+) T cells drive gastrointestinal acute graft-versus-host disease**. *Sci Transl Med* 2021, **13**(576).

49. Ozga AJ, Chow MT, Lopes ME, Servis RL, Di Pilato M, Dehio P, Lian J, Mempel TR, Luster AD: **CXCL10 chemokine regulates heterogeneity of the CD8(+) T cell response and viral set point during chronic infection**. *Immunity* 2022, **55**(1):82-97.e88.

50. Park CO, Fu X, Jiang X, Pan Y, Teague JE, Collins N, Tian T, O'Malley JT, Emerson RO, Kim JH *et al*: **Staged development of long-lived T-cell receptor αβ T(H)17 resident memory T-cell population to Candida albicans after skin infection**. *J Allergy Clin Immunol* 2018, **142**(2):647-662.

51. Herrera-De La Mata S, Ramírez-Suástegui C, Mistry H, Castañeda-Castro FE, Kyyaly MA, Simon H, Liang S, Lau L, Barber C, Mondal M *et al*: **Cytotoxic CD4(+) tissue-resident memory T cells are associated with asthma severity**. *Med* 2023, **4**(12):875-897.e878.

52. Anand N, Lutshumba J, Whitlow M, Abdelaziz MH, Mani R, Suzuki Y: **Deficiency in indoleamine-2, 3-dioxygenase induces upregulation of guanylate binding protein 1 and inducible nitric oxide synthase expression in the brain during cerebral infection with Toxoplasma gondii in genetically resistant BALB/c mice but not in genetically susceptible C57BL/6 mice**. *Microbes Infect* 2022, **24**(3):104908.

53. Lutshumba J, Ochiai E, Sa Q, Anand N, Suzuki Y: **Selective Upregulation of Transcripts for Six Molecules Related to T Cell Costimulation and Phagocyte Recruitment and Activation among 734 Immunity-Related Genes in the Brain during Perforin-Dependent, CD8(+) T Cell-Mediated Elimination of Toxoplasma gondii Cysts**. *mSystems* 2020, **5**(2).

54. Dong J, Chang H-D, Radbruch A: **Epigenetic Imprinting of Immunological Memory**. In: *Epigenetics - A Different Way of Looking at Genetics.* Edited by Doerfler W, Böhm P. Cham: Springer International Publishing; 2016: 53-67.

55. Deng X, Du W, Gasparoni G, Salhab A, Nordström K, Li J, Wagner V, Zhang E, Wachtlin J, Bodo J *et al*: **Methylomes of human CD4 and CD8 memory T lymphocytes reveal tissue-specific epigenetic signatures for maintenance and recall function**. *Immunity & Inflammation* 2025, **1**(1):13.

56. Tsuchiya Y, Namiuchi Y, Wako H, Tsurui H: **A study of CDR3 loop dynamics reveals distinct mechanisms of peptide recognition by T-cell receptors exhibiting different levels of cross-reactivity**. *Immunology* 2018, **153**(4):466-478.

57. Cole DK, Miles KM, Madura F, Holland CJ, Schauenburg AJ, Godkin AJ, Bulek AM, Fuller A, Akpovwa HJ, Pymm PG *et al*: **T-cell receptor (TCR)-peptide specificity overrides affinity-enhancing TCR-major histocompatibility complex interactions**. *J Biol Chem* 2014, **289**(2):628-638.

58. Gleimer M, Wahl AR, Hickman HD, Abi-Rached L, Norman PJ, Guethlein LA, Hammond JA, Draghi M, Adams EJ, Juo S *et al*: **Although divergent in residues of the peptide binding site, conserved chimpanzee Patr-AL and polymorphic human HLA-A*02 have overlapping peptide-binding repertoires**. *J Immunol* 2011, **186**(3):1575-1588.

59. Hamza H, Ghosh M, Löffler MW, Rammensee HG, Planz O: **Identification and relative abundance of naturally presented and cross-reactive influenza A virus MHC class I-restricted T cell epitopes**. *Emerg Microbes Infect* 2024, **13**(1):2306959.

60. Holland CJ, Rizkallah PJ, Vollers S, Calvo-Calle JM, Madura F, Fuller A, Sewell AK, Stern LJ, Godkin A, Cole DK: **Minimal conformational plasticity enables TCR cross-reactivity to different MHC class II heterodimers**. *Sci Rep* 2012, **2**:629.

61. Welsh RM, Selin LK: **No one is naive: the significance of heterologous T-cell immunity**. *Nat Rev Immunol* 2002, **2**(6):417-426.

62. Su LF, Kidd BA, Han A, Kotzin JJ, Davis MM: **Virus-specific CD4(+) memory-phenotype T cells are abundant in unexposed adults**. *Immunity* 2013, **38**(2):373-383.

63. Chaisawangwong W, Wang H, Kouo T, Salathe SF, Isser A, Bieler JG, Zhang ML, Livingston NK, Li S, Horowitz JJ *et al*: **Cross-reactivity of SARS-CoV-2- and influenza A-specific T cells in individuals exposed to SARS-CoV-2**. *JCI Insight* 2022, **7**(18).

64. Grifoni A, Weiskopf D, Ramirez SI, Mateus J, Dan JM, Moderbacher CR, Rawlings SA, Sutherland A, Premkumar L, Jadi RS *et al*: **Targets of T Cell Responses to SARS-CoV-2 Coronavirus in Humans with COVID-19 Disease and Unexposed Individuals**. *Cell* 2020, **181**(7):1489-1501.e1415.

65. Mateus J, Grifoni A, Tarke A, Sidney J, Ramirez SI, Dan JM, Burger ZC, Rawlings SA, Smith DM, Phillips E *et al*: **Selective and cross-reactive SARS-CoV-2 T cell epitopes in unexposed humans**. *Science* 2020, **370**(6512):89-94.

66. Pothast CR, Dijkland RC, Thaler M, Hagedoorn RS, Kester MGD, Wouters AK, Hiemstra PS, van Hemert MJ, Gras S, Falkenburg JHF *et al*: **SARS-CoV-2-specific CD4(+) and CD8(+) T cell responses can originate from cross-reactive CMV-specific T cells**. *Elife* 2022, **11**.

67. Keeton R, Tincho MB, Ngomti A, Baguma R, Benede N, Suzuki A, Khan K, Cele S, Bernstein M, Karim F *et al*: **T cell responses to SARS-CoV-2 spike cross-recognize Omicron**. *Nature* 2022, **603**(7901):488-492.

68. Xiang SD, Gao Q, Wilson KL, Heyerick A, Plebanski M: **A Nanoparticle Based Sp17 Peptide Vaccine Exposes New Immuno-Dominant and Species Cross-reactive B Cell Epitopes**. *Vaccines (Basel)* 2015, **3**(4):875-893.

69. Kover K, Hegre O, Popiela H, Biggs T, Moore WV: **Cross-reactivity of organs in allograft rejection. Comparison of effect of thyroid allografts on established islet allografts**. *Diabetes* 1987, **36**(11):1268-1270.

70. Peereboom ETM, Matern BM, Tomosugi T, Niemann M, Drylewicz J, Joosten I, Allebes WA, van der Meer A, Hilbrands LB, Baas MC *et al*: **T-Cell Epitopes Shared Between Immunizing HLA and Donor HLA Associate With Graft Failure After Kidney Transplantation**. *Front Immunol* 2021, **12**:784040.

71. Picarda E, Bézie S, Usero L, Ossart J, Besnard M, Halim H, Echasserieau K, Usal C, Rossjohn J, Bernardeau K *et al*: **Cross-Reactive Donor-Specific CD8(+) Tregs Efficiently Prevent Transplant Rejection**. *Cell Rep* 2019, **29**(13):4245-4255.e4246.

72. Wang J, Jelcic I, Mühlenbruch L, Haunerdinger V, Toussaint NC, Zhao Y, Cruciani C, Faigle W, Naghavian R, Foege M *et al*: **HLA-DR15 Molecules Jointly Shape an Autoreactive T Cell Repertoire in Multiple Sclerosis**. *Cell* 2020, **183**(5):1264-1281.e1220.

73. Reynolds CJ, Sim MJ, Quigley KJ, Altmann DM, Boyton RJ: **Autoantigen cross-reactive environmental antigen can trigger multiple sclerosis-like disease**. *J Neuroinflammation* 2015, **12**:91.

74. Edwards J, Wilmott JS, Madore J, Gide TN, Quek C, Tasker A, Ferguson A, Chen J, Hewavisenti R, Hersey P *et al*: **CD103(+) Tumor-Resident CD8(+) T Cells Are Associated with Improved Survival in Immunotherapy-Naïve Melanoma Patients and Expand Significantly During Anti-PD-1 Treatment**. *Clin Cancer Res* 2018, **24**(13):3036-3045.

75. Rosato PC, Wijeyesinghe S, Stolley JM, Nelson CE, Davis RL, Manlove LS, Pennell CA, Blazar BR, Chen CC, Geller MA *et al*: **Virus-specific memory T cells populate tumors and can be repurposed for tumor immunotherapy**. *Nature Communications* 2019, **10**(1):567.

76. Chiou SH, Tseng D, Reuben A, Mallajosyula V, Molina IS, Conley S, Wilhelmy J, McSween AM, Yang X, Nishimiya D *et al*: **Global analysis of shared T cell specificities in human non-small cell lung cancer enables HLA inference and antigen discovery**. *Immunity* 2021, **54**(3):586-602.e588.

77. Simoni Y, Becht E, Fehlings M, Loh CY, Koo SL, Teng KWW, Yeong JPS, Nahar R, Zhang T, Kared H *et al*: **Bystander CD8(+) T cells are abundant and phenotypically distinct in human tumour infiltrates**. *Nature* 2018, **557**(7706):575-579.

78. Newman JH, Chesson CB, Herzog NL, Bommareddy PK, Aspromonte SM, Pepe R, Estupinian R, Aboelatta MM, Buddhadev S, Tarabichi S *et al*: **Intratumoral injection of the seasonal flu shot converts immunologically cold tumors to hot and serves as an immunotherapy for cancer**. *Proc Natl Acad Sci U S A* 2020, **117**(2):1119-1128.

79. Sefrin JP, Hillringhaus L, Mundigl O, Mann K, Ziegler-Landesberger D, Seul H, Tabares G, Knoblauch D, Leinenbach A, Friligou I *et al*: **Sensitization of Tumors for Attack by Virus-Specific CD8+ T-Cells Through Antibody-Mediated Delivery of Immunogenic T-Cell Epitopes**. *Front Immunol* 2019, **10**:1962.

80. Fusciello M, Ylösmäki E, Feola S, Uoti A, Martins B, Aalto K, Hamdan F, Chiaro J, Russo S, Viitala T *et al*: **A novel cancer vaccine for melanoma based on an approved vaccine against measles, mumps, and rubella**. *Mol Ther Oncolytics* 2022, **25**:137-145.

81. Zhang Y, Gabere M, Taylor MA, Simoes CC, Dumbauld C, Barro O, Tesfay MZ, Graham AL, Ferdous KU, Savenka AV *et al*: **Repurposing live attenuated trivalent MMR vaccine as cost-effective cancer immunotherapy**. *Front Oncol* 2022, **12**:1042250.

82. Ragone C, Manolio C, Cavalluzzo B, Mauriello A, Tornesello ML, Buonaguro FM, Castiglione F, Vitagliano L, Iaccarino E, Ruvo M *et al*: **Identification and validation of viral antigens sharing sequence and structural homology with tumor-associated antigens (TAAs)**. *J Immunother Cancer* 2021, **9**(5).

83. Quezada SA, Simpson TR, Peggs KS, Merghoub T, Vider J, Fan X, Blasberg R, Yagita H, Muranski P, Antony PA *et al*: **Tumor-reactive CD4(+) T cells develop cytotoxic activity and eradicate large established melanoma after transfer into lymphopenic hosts**. *J Exp Med* 2010, **207**(3):637-650.

84. Oh DY, Kwek SS, Raju SS, Li T, McCarthy E, Chow E, Aran D, Ilano A, Pai CS, Rancan C *et al*: **Intratumoral CD4(+) T Cells Mediate Anti-tumor Cytotoxicity in Human Bladder Cancer**. *Cell* 2020, **181**(7):1612-1625.e1613.

85. Anand N, Peh KH, Kolesar JM: **Macrophage Repolarization as a Therapeutic Strategy for Osteosarcoma**. *Int J Mol Sci* 2023, **24**(3).

86. Wu S, Anand N, Guo Z, Li M, Santiago Figueroa M, Jung L, Kelly S, Franses JW: **Bridging Immune Evasion and Vascular Dynamics for Novel Therapeutic Frontiers in Hepatocellular Carcinoma**. *Cancers (Basel)* 2025, **17**(11).

87. Ji Q, Perchellet A, Goverman JM: **Viral infection triggers central nervous system autoimmunity via activation of CD8+ T cells expressing dual TCRs**. *Nature Immunology* 2010, **11**(7):628-634.

88. Vanderlugt CL, Begolka WS, Neville KL, Katz-Levy Y, Howard LM, Eagar TN, Bluestone JA, Miller SD: **The functional significance of epitope spreading and its regulation by co-stimulatory molecules**. *Immunol Rev* 1998, **164**:63-72.

89. Vanderlugt CJ, Miller SD: **Epitope spreading**. *Curr Opin Immunol* 1996, **8**(6):831-836.

90. Pande H, Campo K, Shanley JD, Creeger ES, Artishevsky A, Gallez-Hawkins G, Zaia JA: **Characterization of a 52K protein of murine cytomegalovirus and its immunological cross-reactivity with the DNA-binding protein ICP36 of human cytomegalovirus**. *J Gen Virol* 1991, **72 ( Pt 6)**:1421-1427.

91. Chen YF, Hsieh AH, Wang LC, Yu KH, Kuo CF: **Cytomegalovirus-Associated Autoantibody against TAF9 Protein in Patients with Systemic Lupus Erythematosus**. *J Clin Med* 2021, **10**(16).

92. Crowl JT, Heeg M, Ferry A, Milner JJ, Omilusik KD, Toma C, He Z, Chang JT, Goldrath AW: **Tissue-resident memory CD8(+) T cells possess unique transcriptional, epigenetic and functional adaptations to different tissue environments**. *Nat Immunol* 2022, **23**(7):1121-1131.

93. Fernandez SA, Pelaez-Prestel HF, Fiyouzi T, Gomez-Perosanz M, Reiné J, Reche PA: **Tetanus-diphtheria vaccine can prime SARS-CoV-2 cross-reactive T cells**. *Front Immunol* 2024, **15**:1425374.

94. Hasan F, Chiu Y, Shaw RM, Wang J, Yee C: **Hypoxia acts as an environmental cue for the human tissue-resident memory T cell differentiation program**. *JCI Insight* 2021, **6**(10).

95. Zhu HX, Yang SH, Gao CY, Bian ZH, Chen XM, Huang RR, Meng QL, Li X, Jin H, Tsuneyama K *et al*: **Targeting pathogenic CD8(+) tissue-resident T cells with chimeric antigen receptor therapy in murine autoimmune cholangitis**. *Nat Commun* 2024, **15**(1):2936.

96. Li J, Reinke S, Shen Y, Schollmeyer L, Liu YC, Wang Z, Hardt S, Hipfl C, Hoffmann U, Frischbutter S *et al*: **A ubiquitous bone marrow reservoir of preexisting SARS-CoV-2-reactive memory CD4(+) T lymphocytes in unexposed individuals**. *Front Immunol* 2022, **13**:1004656.

97. Lutter L, Roosenboom B, Brand EC, Ter Linde JJ, Oldenburg B, van Lochem EG, Horjus Talabur Horje CS, van Wijk F: **Homeostatic Function and Inflammatory Activation of Ileal CD8(+) Tissue-Resident T Cells Is Dependent on Mucosal Location**. *Cell Mol Gastroenterol Hepatol* 2021, **12**(5):1567-1581.

98. Barsch M, Salié H, Schlaak AE, Zhang Z, Hess M, Mayer LS, Tauber C, Otto-Mora P, Ohtani T, Nilsson T *et al*: **T-cell exhaustion and residency dynamics inform clinical outcomes in hepatocellular carcinoma**. *J Hepatol* 2022, **77**(2):397-409.

99. Kok L, Dijkgraaf FE, Urbanus J, Bresser K, Vredevoogd DW, Cardoso RF, Perié L, Beltman JB, Schumacher TN: **A committed tissue-resident memory T cell precursor within the circulating CD8+ effector T cell pool**. *J Exp Med* 2020, **217**(10).

100. Tagkareli S, Salagianni M, Galani IE, Manioudaki M, Pavlos E, Thanopoulou K, Andreakos E: **CD103 integrin identifies a high IL-10-producing FoxP3(+) regulatory T-cell population suppressing allergic airway inflammation**. *Allergy* 2022, **77**(4):1150-1164.

101. Del Campo J, Bouley J, Chevandier M, Rousset C, Haller M, Indalecio A, Guyon-Gellin D, Le Vert A, Hill F, Djebali S *et al*: **OVX836 Heptameric Nucleoprotein Vaccine Generates Lung Tissue-Resident Memory CD8+ T-Cells for Cross-Protection Against Influenza**. *Front Immunol* 2021, **12**:678483.

102. Slütter B, Pewe LL, Lauer P, Harty JT: **Cutting edge: rapid boosting of cross-reactive memory CD8 T cells broadens the protective capacity of the Flumist vaccine**. *J Immunol* 2013, **190**(8):3854-3858.

103. Hu C, Wang Z, Ren L, Hao Y, Zhu M, Jiang H, Wang S, Li D, Shao Y: **Pre-existing anti-HCoV-OC43 immunity influences the durability and cross-reactivity of humoral response to SARS-CoV-2 vaccination**. *Front Cell Infect Microbiol* 2022, **12**:978440.

104. Woodworth JS, Clemmensen HS, Battey H, Dijkman K, Lindenstrøm T, Laureano RS, Taplitz R, Morgan J, Aagaard C, Rosenkrands I *et al*: **A Mycobacterium tuberculosis-specific subunit vaccine that provides synergistic immunity upon co-administration with Bacillus Calmette-Guérin**. *Nat Commun* 2021, **12**(1):6658.

105. Fluckiger A, Daillère R, Sassi M, Sixt BS, Liu P, Loos F, Richard C, Rabu C, Alou MT, Goubet AG *et al*: **Cross-reactivity between tumor MHC class I-restricted antigens and an enterococcal bacteriophage**. *Science* 2020, **369**(6506):936-942.

106. Helman SR, Stevanovic S, Campbell TE, Kwong MLM, Doran SL, Faquin WC, Hinrichs CS: **Human Papillomavirus T-Cell Cross-reactivity in Cervical Cancer: Implications for Immunotherapy Clinical Trial Design**. *JAMA Netw Open* 2018, **1**(3):e180706.

107. Dong C, Lin L, Du J: **Characteristics and sources of tissue-resident memory T cells in psoriasis relapse**. *Curr Res Immunol* 2023, **4**:100067.

108. Yokoi T, Murakami M, Kihara T, Seno S, Arase M, Wing JB, Søndergaard JN, Kuwahara R, Minagawa T, Oguro-Igashira E *et al*: **Identification of a unique subset of tissue-resident memory CD4(+) T cells in Crohn's disease**. *Proc Natl Acad Sci U S A* 2023, **120**(1):e2204269120.

109. Li C, Zhu B, Son YM, Wang Z, Jiang L, Xiang M, Ye Z, Beckermann KE, Wu Y, Jenkins JW *et al*: **The Transcription Factor Bhlhe40 Programs Mitochondrial Regulation of Resident CD8(+) T Cell Fitness and Functionality**. *Immunity* 2019, **51**(3):491-507.e497.

110. Knudson CJ, Férez M, Alves-Peixoto P, Erkes DA, Melo-Silva CR, Tang L, Snyder CM, Sigal LJ: **Mechanisms of Antiviral Cytotoxic CD4 T Cell Differentiation**. *J Virol* 2021, **95**(19):e0056621.

111. Hao Q, Kundu S, Shetty S, Tucker TA, Idell S, Tang H: **Inducible general knockout of Runx3 profoundly reduces pulmonary cytotoxic CD8(+) T cells with minimal effect on outcomes in mice following influenza infection**. *Front Immunol* 2022, **13**:1011922.

112. Boyman O, Hefti HP, Conrad C, Nickoloff BJ, Suter M, Nestle FO: **Spontaneous development of psoriasis in a new animal model shows an essential role for resident T cells and tumor necrosis factor-alpha**. *J Exp Med* 2004, **199**(5):731-736.

113. Clark RA, Chong B, Mirchandani N, Brinster NK, Yamanaka K, Dowgiert RK, Kupper TS: **The vast majority of CLA+ T cells are resident in normal skin**. *J Immunol* 2006, **176**(7):4431-4439.

114. Mizukawa Y, Shiohara T: **Trauma-localized fixed drug eruption: involvement of burn scars, insect bites and venipuncture sites**. *Dermatology* 2002, **205**(2):159-161.

115. Shiohara T, Ushigome Y, Kano Y, Takahashi R: **Crucial Role of Viral Reactivation in the Development of Severe Drug Eruptions: a Comprehensive Review**. *Clin Rev Allergy Immunol* 2015, **49**(2):192-202.

116. Siracusa F, McGrath MA, Maschmeyer P, Bardua M, Lehmann K, Heinz G, Durek P, Heinrich FF, Mashreghi MF, Chang HD *et al*: **Nonfollicular reactivation of bone marrow resident memory CD4 T cells in immune clusters of the bone marrow**. *Proc Natl Acad Sci U S A* 2018, **115**(6):1334-1339.

117. Nelson CE, Foreman TW, Fukutani ER, Kauffman KD, Sakai S, Fleegle JD, Gomez F, Gould ST, Le Nouën C, Liu X *et al*: **IL-10 suppresses T cell expansion while promoting tissue-resident memory cell formation during SARS-CoV-2 infection in rhesus macaques**. *PLoS Pathog* 2024, **20**(7):e1012339.

118. Luo S, Zhang P, Wang Y, Huang Y, Ma X, Deng Q, Zou P, Wang C, Zhang L, Li Y *et al*: **Adenoviruses vectored hepatitis C virus vaccine cocktails induce broadly specific immune responses against multi-genotypic HCV in mice**. *Biomed Pharmacother* 2024, **170**:115901.

119. Jiao W, Long KD, Young T, Muntnich CB, Rey AP, Khwajazadah M, Wang JH, Mohan V, Rogers K, Valena A *et al*: **Immunogenomic landscape of T cell repertoire after human lung transplantation and its clinical significance**. *medRxiv* 2025.

120. Jiao W, Martinez M, Muntnich CB, Zuber J, Parks C, Obradovic A, Tian G, Wang Z, Long KD, Waffarn E *et al*: **Dynamic establishment of recipient resident memory T cell repertoire after human intestinal transplantation**. *EBioMedicine* 2024, **101**:105028.

121. Dravid P, Murthy S, Attia Z, Cassady C, Chandra R, Trivedi S, Vyas A, Gridley J, Holland B, Kumari A *et al*: **Phenotype and fate of liver-resident CD8 T cells during acute and chronic hepacivirus infection**. *PLoS Pathog* 2023, **19**(10):e1011697.

122. Zimmer CL, von Seth E, Buggert M, Strauss O, Hertwig L, Nguyen S, Wong AYW, Zotter C, Berglin L, Michaëlsson J *et al*: **A biliary immune landscape map of primary sclerosing cholangitis reveals a dominant network of neutrophils and tissue-resident T cells**. *Sci Transl Med* 2021, **13**(599).

123. Abou-Daya KI, Tieu R, Zhao D, Rammal R, Sacirbegovic F, Williams AL, Shlomchik WD, Oberbarnscheidt MH, Lakkis FG: **Resident memory T cells form during persistent antigen exposure leading to allograft rejection**. *Sci Immunol* 2021, **6**(57).

124. Schøller AS, Fonnes M, Nazerai L, Christensen JP, Thomsen AR: **Local Antigen Encounter Is Essential for Establishing Persistent CD8(+) T-Cell Memory in the CNS**. *Front Immunol* 2019, **10**:351.

125. Altendorfer B, Unger MS, Poupardin R, Hoog A, Asslaber D, Gratz IK, Mrowetz H, Benedetti A, de Sousa DMB, Greil R *et al*: **Transcriptomic Profiling Identifies CD8(+) T Cells in the Brain of Aged and Alzheimer's Disease Transgenic Mice as Tissue-Resident Memory T Cells**. *J Immunol* 2022, **209**(7):1272-1285.
